# On the analysis of metabolite quantitative trait loci: Impact of different data transformations and study designs

**DOI:** 10.1126/sciadv.adp4532

**Published:** 2025-04-11

**Authors:** Sanghun Lee, Rachel S. Kelly, Kevin M. Mendez, Dmitry Prokopenko, Georg Hahn, Sharon M. Lutz, Juan C. Celedón, Clary B. Clish, Scott T. Weiss, Christoph Lange, Jessica A. Lasky-Su, Julian Hecker, NHLBI Trans-Omics for Precision Medicine (TOPMed) Consortium

**Affiliations:** ^1^ Department of Medical Consilience, Division of Medicine, Graduate School, Dankook University, Yongin-si, South Korea.; ^2^ Channing Division of Network Medicine, Brigham and Women’s Hospital, Harvard Medical School, Boston, MA, USA.; ^3^ Department of Biostatistics, Harvard T.H. Chan School of Public Health, Boston, MA, USA.; ^4^ Genetics and Aging Research Unit and the McCance Center for Brain Health, Department of Neurology, Massachusetts General Hospital, Boston, MA, USA.; ^5^ Department of Population Medicine, Harvard Medical School and Harvard Pilgrim Healthcare Institute, Boston, MA, USA.; ^6^ Division of Pediatric Pulmonary Medicine, Department of Pediatrics, University of Pittsburgh, Pittsburgh, PA, USA.; ^7^ Metabolomics Platform, Broad Institute, Cambridge, MA, USA.

## Abstract

Metabolomic genome-wide association studies (mGWASs), or metabolomic quantitative trait locus (metQTL) analyses, are gaining growing attention. However, robust methods and analysis guidelines, vital to address the complexity of metabolomic data, remain to be established. Here, we use whole-genome sequencing and metabolomic data from two independent studies to compare different approaches. We adopted three popular data transformation methods for metabolite levels—(i) log_10_ transformation, (ii) rank inverse normal transformation, and (iii) a fully adjusted two-step procedure—and compared population-based versus family-based analysis approaches. For validation, we performed permutation-based testing, Huber regression, and independent replication analysis. Simulation studies were used to illustrate the observed differences between data transformations. We demonstrate the advantages and limitations of popular analytic strategies used in mGWASs where especially low-frequency variants in combination with a skewed metabolite measurement distribution can lead to potentially false-positive metQTL findings. We recommend the rank inverse normal transformation or robust test statistics such as in family-based association tests as reliable approaches for mGWASs.

## Teaser

Selecting robust analytical strategies for metQTL analyses improves reliability and reproducibility in metabolomic studies.

## INTRODUCTION

Genome-wide association studies (GWASs) are commonly used to investigate the genetic basis of complex traits or diseases (*1*). In recent years, there has been a growing interest in the use of GWASs to explore the genetic basis of the metabolome, the complete set of small molecules, or metabolites, present in an organism (*2*–*12*). These analyses are commonly referred to as metabolomic GWASs (mGWASs) or metabolomic quantitative trait locus (metQTL) studies. Metabolites are the downstream products of complex interactions between underlying genetic susceptibility and environmental exposures, and they can provide important insights into the biochemical pathways and physiological processes that result in changes in a phenotype (*5*, *13*).

One of the main challenges in conducting mGWAS/metQTL studies is the skewness of metabolite measurement distributions, impeding the application of standard statistical approaches, which require at least some distributional assumptions (*14*, *15*). To address the issue of distributional skewness in statistical data analysis, there are three common approaches: (i) data transformations to meet the distributional assumptions of regression analyses (*16*–*19*), (ii) regression approaches that are robust to outliers such as Huber regression (*20*), or (iii) permutation-based testing that requires only minimal distributional assumptions (*21*, *22*). However, to date, there has not been a systematic comparison of these approaches in the context of mGWAS.

Data transformations such as log transformation or rank inverse normal transformation can help to reduce skewness and therefore allow the application of existing and standard regression analyses. Hence, they are commonly used in metabolomics (*2*, *8*–*11*). Robust approaches, for example, Huber regression, reduce sensitivity toward data outliers and have been successfully implemented and used in the analysis of transcriptomics and epigenetics data (*23*, *24*), but have not yet been widely applied to metabolomic data analysis. Last, permutation-based testing does not require strong distributional assumptions but is computationally challenging, especially given the high dimensionality of metabolomic data that results in a large number of statistical tests. The most suitable approach for dealing with skewness in metabolomic data may depend on the specific experimental context and the goals of the analysis. Moreover, most mGWASs use unrelated samples only, and potential confounding is routinely adjusted for by using the principal components (PCs) of genetic ancestry (*5*, *8*, *10*). However, it is unclear to what extent this affects findings in the context of metQTL studies. Therefore, careful consideration and evaluation of the available methods are necessary to ensure reliable and meaningful interpretation of metabolomic data.

Using two large independent, diverse multi-omic studies of children with asthma and their parents, we are in a unique position to (i) investigate the dependency and sensitivity of mGWAS/metQTL results to different data transformations, robust regression models, and permutation-based testing and (ii) compare population-based analysis with family-based analysis, which is completely robust to genetic confounding and misspecification of the phenotype distribution (*25*). Together, these results will help to inform the choice of methods for future mGWAS analyses, as interest continues to grow in the relationship between genetic variants and metabolite levels.

## RESULTS

### Study subjects

**Fig. 1.**
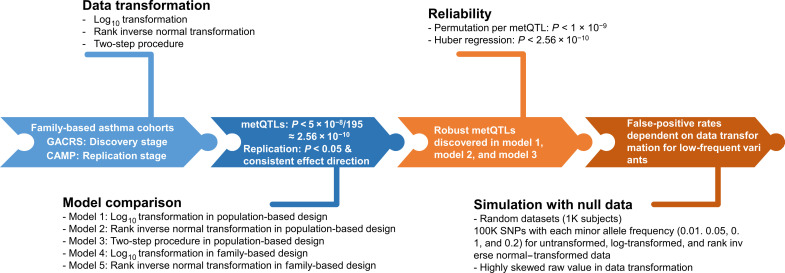
Workflow. Significant metQTL-associations were identified on the basis of an asymptotic *P* value of <2.56 × 10^−10^, corresponding to the standard genome-wide significance level of 5 × 10^−8^ additionally adjusted for 195 metabolites tested, using a Bonferroni correction. Permutation-based *P* values were declared as significant using *P* < 10^−9^, taking into account the maximum number of permutations of 10^9^ in PLINK2.

Our analyses are based on participants from two independent studies: the Genetics of Asthma in Costa Rica Study (GACRS) and the Childhood Asthma Management Program (CAMP). The study protocol and subject recruitment details for both studies have been published previously (*26*–*29*). The workflow of our investigations is visualized in Fig. 1. After merging quality-controlled whole-genome sequencing (WGS) and metabolite data, we obtained 1021 unrelated GACRS and 712 unrelated CAMP children with both WGS and metabolomic profiling data who were included in the population-based analysis. For the family-based analysis, we included 897 GACRS trios and 340 CAMP trios with WGS data for all three family members and offspring metabolite data available (Table 1).

**Table 1.** Demographic characteristics of study subjects in GACRS and CAMP. Mean ± SD or number (%) is shown.

	GACRS children	GACRS offspring in trios^*^	CAMP children	CAMP offspring in trios^*^
Number	1021	897	712	340
Male, *n* (%)	608 (59.55)	521 (58.08)	432 (60.67)	223 (65.59)
Age, years (SD)	9.25 ± 1.87	9.22 ± 1.86	13.07 ± 2.16	12.94 ± 2.14
Body mass index, kg/m^2^ (SD)	18.30 ± 3.79	18.30 ± 3.88	21.63 ± 4.63	21.62 ± 4.59
Self-reported ethnicity				
African, n (%)			89 (12.50)	36 (10.59)
European, n (%)			494 (69.38)	248 (72.94)
Hispanic, n (%)	1021 (100.0)	897 (100.0)	69 (9.69)	27 (7.94)
Other, n (%)			60 (8.43)	29 (8.53)

*Data for offspring in trio.

The GACRS discovery population was slightly younger than CAMP and had a lower body mass index (BMI) (age at collection for metabolite data). All GACRS subjects were Hispanic, but most of the CAMP participants (69.38%) were of European ancestry, with around 10% Hispanic. A total of 195 metabolites of known structural identity were measured in both cohorts on the HILIC (hydrophilic interaction liquid chromatography)–positive platform, covering a broad range of the metabolome, including amino acids, peptides, carbohydrates, energy intermediates, lipids, nucleotides, cofactors and vitamins, and xenobiotics.

### Five different metQTL analysis approaches (models 1 to 5)

We adopted five different metQTL analysis approaches, incorporating different data transformations and population-based (models 1 to 3) and family-based testing (models 4 and 5). The different analysis approaches are described below and summarized in Table 2.

**Table 2.** Genetic association analysis of metabolite data using five different approaches.

Regression	Outcome	Covariates	Model type and software
Model 1	Log_10_-transformed metabolite levels	Age + Sex + BMI + genetic ancestry PC1:10	Population based; PLINK2
Model 2	Rank inverse normal–transformed metabolite levels	Age + Sex + BMI + genetic ancestry PC1:10	Population based; PLINK2
Model 3	Two-step procedure: Untransformed metabolite levels regressed on covariates, rank inverse normal transformation of residuals as outcome in second step regression with same covariates + genetic variant as predictors	Age + Sex + BMI + genetic ancestry PC1:10	Population based; PLINK2
Model 4	Log_10_-transformed metabolite levels	Age + Sex + BMI^*^	Family based; FBAT
Model 5	Rank inverse normal–transformed metabolite levels	Age + Sex + BMI^*^	Family based; FBAT

*Regression adjustment for covariates; residuals considered as the outcome in FBAT analysis.

For the population-based approach, the metabolite levels in model 1 were log_10_ transformed and Pareto scaled. Here, we note that Pareto scaling is a commonly used approach to scale metabolite data, but it does not affect the results in our studies for model 1, as it just corresponds to a linear transformation of the outcome data. The regression model included age, sex, BMI, and the top 10 PCs of genetic ancestry as additional covariates. For model 2, metabolite levels were rank inverse normal transformed and adjusted for the same covariates in the regression model. We applied a fully adjusted two-step approach in model 3 (*30*). Here, untransformed metabolite levels were first regressed on the same covariates as in models 1 and 2. Subsequently, the corresponding residuals underwent a rank inverse normal transformation and were incorporated into a second-step regression analysis using the same covariates and the genetic variant of interest as predictors. The coefficient of the genetic variant was tested for significance to obtain the association *P* value.

For the family-based analysis, we applied the family-based association test (FBAT) framework (*25*, *31*). FBAT is a conditional score test, conditioning on the sufficient statistic for parental genotypes and the observed phenotype. The outcome variable was defined as the residuals obtained after regressing transformed metabolite levels on age, sex, and BMI. No adjustment for PCs of genetic ancestry was performed. Two different data transformations were considered: log_10_ transformation (model 4) and rank inverse normal transformation (model 5).

The association between a single-nucleotide polymorphism (SNP) and a metabolite in a given model was considered significant if the corresponding asymptotic *P* value was less than *P* < 5 × 10^−8^/195 ≒ 2.56 × 10^−10^, which represents the standard genome-wide significance level adjusted for the number of metabolites tested using a Bonferroni correction.

### Genome-wide significant SNP-metabolite associations in population-based analyses

In general, the population-based analyses identified more significant SNP-metabolite associations, which we refer to as metQTL-associations, than the family-based analyses (Table 3). Within the population-based analyses, a larger number of significant independent metQTL-associations, *n* = 159, were identified using the log_10_-transformed metabolite levels (model 1) compared to only 54 using the rank inverse normal–transformed metabolite levels (model 2). Notably, 1977 significant independent metQTL-associations were identified with the two-step procedure (model 3), linked to a relatively small number of metabolites (*n* = 46) (Table 3).

**Table 3.** Number of SNP-metabolite associations (metQTL-associations) identified across the five different models.

Study design	Model	Unique metabolites involved in significant metQTL-associations^*^	Unique SNPs involved in significant metQTL-associations^*^	metQTL-associations^*^	*n* (%), metQTL-associations in permutation test; *P* < 1 × 10^−9^	*n* (%), permutation test metQTL-association common to the GACRS models	*n* (%), metQTL-associations in Huber test; *P* < 2.56 × 10^−10^	*n* (%), Huber test metQTL-association common to the GACRS models	Missing SNPs involved in significant metQTL-associations in CAMP^†^	*n* (%), metQTL-associations replicated in CAMP^‡^
Population-based study	Model 1	52	137	159	51 (32.08)	49 (96.08) in model 2	49 (30.82)	49 (100.0) in model 2	46	43 (38.05%)
						48 (94.12) in model 3		49 (100.0) in model 3		
	Model 2	42	44	54	53 (98.15)	49 (92.45) in model 1	53 (98.15)	49 (92.45) in model 1	2	43 (82.69%)
						52 (98.11) in model 3		52 (98.11) in model 3		
	Model 3	46	1968	1977	52 (2.63)	49 (94.23) in model 1	54 (2.73)	49 (90.74) in model 1	871	70 (6.33%)
						52 (100.0) in model 2		52 (96.30) in model 2		
Family-based study	Model 4	12	10	12	–	–	–	–	0	12 (100.0%)
	Model 5	15	14	16	–	–	–	–	0	13 (81.25%)

*A significant metQTL-association was defined by a corresponding Bonferroni-corrected asymptotic *P* < 2.56 × 10^−10^.

†Number of cases where the genetic variant identified as metQTL-association in the GACRS discovery analysis was not available in CAMP for replication.

‡Replication is defined as an association *P* < 5% in CAMP with a consistent direction of effect. The replication rate in percentages is based on the number of metQTL-associations available for replication.

### Validation of findings in permutation-based testing, Huber regression, and replication in CAMP

To disentangle the observed differences in the number of metQTL-associations across models, we reran the significant metQTL-associations from models 1 to 3 using permutation-based testing and Huber regression analysis. We considered all models 1 to 3 here because data transformations do not only aim to improve distribution characteristics but can also alter the power of the analysis (*19*). Because the distribution of metabolite levels can be skewed, asymptotic *P* values in linear regression models can produce false-positive findings. The applied data transformation approaches attempt to reduce this problem, but the sufficiency of this approach in this context is unknown. Permutation-based testing has the advantage that it requires weaker assumptions about the data distribution. The limitation here is the computational burden, limiting the number of permutations by 1 × 10^9^ (in the PLINK2 software) and therefore restricting the results of permutation-based testing to a minimum permutation *P* value of 1 × 10^−9^. In the following, using a slight simplification, we considered an empirical *P* value of <1 × 10^−9^ as additional evidence for the metQTL-associations representing a true signal and not a statistical artifact.

Table 3 shows that model 2 outperformed models 1 and 3 in terms of validation via permutation-based testing and Huber regression, with >98% of metQTL-associations additionally found to be significant via these approaches (compared to ~30% and ~2% in models 1 and 3, respectively). The results suggested that across the three approaches, permutation-based testing and Huber regression were largely identifying the same metQTL-associations, as the consistency between the permutation-based results and Huber results exceeded >90% across models 1 to 3. The significant metQTL-associations identified using the permutation-based tests for each model are provided in tables S1 to S3.

Further evidence for the detection of a true biological finding was provided by the replication rate when rerunning analyses in the CAMP cohort. Again, the metQTL-associations identified in model 2 using rank inverse normal–transformed values were more likely to be replicated in CAMP (82.69% of metQTL-associations replicated) than results based on log_10_ transformations (model 1, 38.05% replicated) and the two-step procedure (model 3, 6.33% replicated) (Table 3). We note that the lack of replication does not imply that the discovered signal is a false positive, and other explanations include the lack of power and different characteristics of the CAMP cohort compared to GACRS. Together, the results in Table 3 illustrate that the association analysis findings based on model 2 (rank inverse normal transformation) provide significantly more robust results and higher replication rates than models 1 and 3. In conclusion, a substantial fraction of metQTL-associations identified by models 1 and 3 are potentially false-positive findings.

### Minor allele frequency as the driving factor underlying potentially false-positive findings in GACRS

**Fig. 2.**
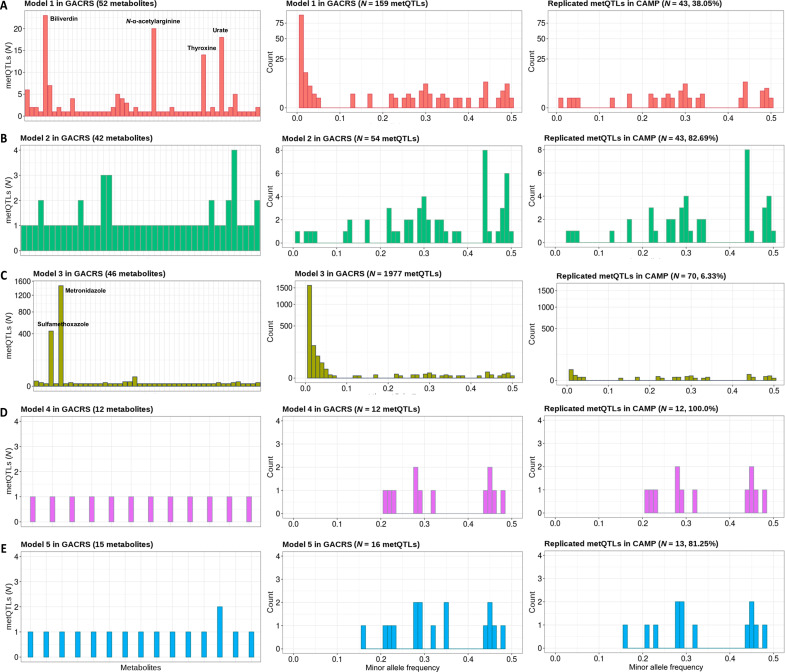
Results of metQTL-associations based on each model (A: model 1, B: model 2, C: model 3, D: model 4, and E: model 5). Left: Number of SNP-metabolite associations (metQTL-associations) identified for each metabolite, reported separately for each model (**A** to **E**). Middle: The MAF distribution of the metQTL-associations in the GACRS discovery population, reported separately for each model. Right: The MAF distribution of the metQTL-associations replicated in CAMP, reported separately for each model.

We next investigated the driving factors underlying these potential false-positive associations. Figure 2 (A to E) illustrates the minor allele frequency (MAF) distribution of the SNPs involved in the identified metQTL-associations and the number of associated independent metQTL-associations with each metabolite for all models. We observed substantial differences between the MAF distributions of the identified metQTL-associations based on the different models. The analysis based on model 1 revealed a large fraction of low-frequency genetic variants (MAF < 5%) involved in metQTL-associations, which were not replicated in CAMP. This contrasts with the results based on model 2 (Fig. 2B). The metabolites associated with low-frequency SNPs (biliverdin, *N*-α-acetylarginine, urate, and thyroxine) in model 1 are the top 4 metabolites in terms of skewness based on log_10_-transformed levels (table S4), whereas the normalization approach in model 2 eliminated skewness and therefore also these spurious associations (Fig. 2, A and B).

The metQTL-associations in model 3 were mainly driven by two metabolites, “metronidazole” and “sulfamethoxazole” (1906 of 1977 associations, ~96%; Fig. 2C), which are the top 2 metabolites in skewness based on untransformed metabolite levels (table S4), leading to strong spurious correlations with the covariates in the second-step regression. This, in turn, leads to skewness in the association analysis between the SNP and the corresponding residuals. We investigated this observation in more detail in the simulation studies described below.

Overall, MAF was the main factor driving the difference in the number of metQTL-associations identified among models in the population-based analysis. In the following section, we describe simulation studies that demonstrate and illustrate these findings further.

### Type 1 error simulation studies

We performed two simulation studies to illustrate and support the conclusions and findings from the real data analysis. The simulation studies used the observed metabolite levels in GACRS data.

The first simulation study investigated type 1 error rates for standard linear regression, Huber regression, and FBAT statistics when analyzing metabolites using untransformed, log-transformed, and rank inverse normal–transformed data. For each of the 195 metabolites included in our analysis, we performed a set of simulations in which we generated simulated metabolite outcomes by randomly sampling with replacement from the observed data (sample size *n* = 1000). We simulated independent SNPs for four different MAFs (1, 5, 10, and 20%, 10,000 simulated SNPs each) and applied all three methods to investigate associations between SNPs and simulated metabolite levels. Because metabolites and SNPs were drawn independently, no true associations were present in the simulated data, enabling the analysis of type 1 error rates.

In the second set of simulations, we extracted the observed untransformed metabolite levels for the metabolite metronidazole as well as the covariates used in the real data analysis (sample size *n* = 1051). This metabolite is described by a highly skewed distribution with outlier values and showed a potentially increased number of false positives in the data analysis. We simulated corresponding independent SNPs with six different MAFs (1 to 20%, 10,000 simulated SNPs each) and analyzed the associations with the simulated metabolite using the two-step approach. Here, we considered untransformed and log-transformed data in the first regression step of the two-step approach.

**Fig. 3.**
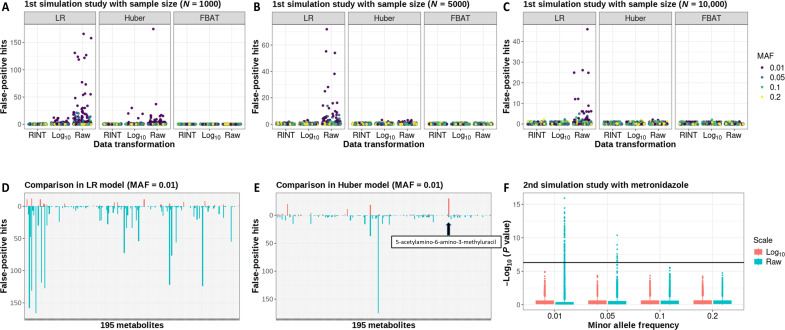
Results of the two simulation studies. Plots (**A**), (**B**), and (**C**) present the results of the first simulation study with an increasing sample size. These plots show the number of false-positive associations for linear regression (LR), Huber regression (Huber), and FBAT among the 195 simulated metabolites, stratified by data transformation and MAF. False-positive findings were defined on the basis of an association *P* value with *P* < 0.05/10,000. As the sample size increases, the number of false-positive associations decreases, although rare allele frequency contributes to an increase in false positives. RINT, rank inverse normal transformation. Plots (**D**) and (**E**) illustrate the differences in the number of false-positive associations for the same metabolite depending on whether data transformation was applied (first simulation study). In general, untransformed metabolite levels result in more false-positive findings compared to log_10_-transformed data. However, certain metabolites, such as 5-acetylamino-6-amino-3-methyluracil, exhibit higher numbers of false positives after a log_10_ transformation. Plot (**F**) shows the results of the second simulation study, which focused on metabolite levels for metronidazole. It displays *P* value distribution from the two-step approach, stratified by data transformation and MAF. The black line represents the Bonferroni-corrected *P* value (0.05/10,000), defining false-positive findings.

The results of the first simulation study demonstrated that standard linear regression shows an increased false-positive rate for metabolites with skewed distributions when the analysis is performed using the untransformed and log-transformed data (Fig. 3A and table S4). In particular, the inflation arises for low-frequency SNPs. This is in line with the observations in our real data analysis and is expected because the sparsity of the SNP data in combination with nonnormal outcomes can lead to inaccurate asymptotic *P* values. In contrast, Huber regression provided more accurate false-positive rates. FBAT showed strongly robust type 1 error control, regardless of MAF and data transformation. Repeating the simulations with larger sample sizes (*n* = 5000 and 10,000) showed that the inflated type 1 error rates are a function of the relation between MAF and sample size, reducing the type 1 error rates compared to the sample size of *n* = 1000 (Fig. 3, B and C, and table S5). We note that the log transformation overall reduces the occurrence of false-positive findings, but for certain metabolites, the type 1 error rate was higher using the log transformation compared to untransformed data (Fig. 3, D and E, and table S5).

The second simulation study showed that highly skewed metabolite data in the first step of the two-step approach can lead to scenarios where the rank inverse normal–transformed residuals are highly associated with the covariates when performing the regression in the second step. By the Frisch-Waugh-Lovell theorem (*32*, *33*), multiple variable regression incorporating the SNP and covariates is equivalent to first regressing the outcome and SNP on the covariates and consequently regressing the outcome residuals on the SNP residuals. Because the outcome is highly associated with the covariates in this regression, the resulting outcome residuals obtain a highly skewed distribution. This leads again to false-positive findings for low-frequency SNPs (Fig. 3F). When using the log transformation to reduce skewness before applying the two-step approach, the type 1 error rate was controlled for metronidazole.

### The performance of family-based analyses in mGWASs

The family-based analyses identified a much smaller number of metQTL-associations than any of the population-based models and appeared to be less dependent on the transformation approach (*n* = 12 in model 4 versus *n* = 16 in model 5). The level of replication in CAMP was high and similar between the two normalization approaches (model 4: 100% and model 5: 81.25%; Table 3). While the reduced number of identified metQTL-associations is likely explained by the reduced power and sample size of this trio-based analysis, the consistency of results across both data transformations is a feature of the FBAT statistic. The nonparametric FBAT statistics for association between SNPs and metabolites given the observed metabolite levels do not require strong model assumptions regarding the phenotype distribution (*25*). Moreover, the robust variance estimator of FBAT provides additional robustness against skewed distributions and data outliers. Hence, FBAT performed well even with low MAF variants.

### Concordance of metQTL-associations across all models

Across the three population-based models in GACRS, 41 metQTL-associations were common to all three, of which 34 (82.9%) were replicated in CAMP (fig. S1). The findings from the family-based analyses were also among these 41 associations.

Among the significant results based on the permutation-based testing, there was a much higher level of agreement across the three models (fig. S1), where a total of 47 metQTL-associations were overlapping after considering linkage disequilibrium between SNPs in metQTL-associations (Table 4). On the basis of a comprehensive search of published metQTL-associations in the GWAS catalog (*34*), we determined that 38 (80.1%) metQTL-associations have previously been reported in the literature, most of which were replicated in CAMP (Table 4). Many of those that have not been previously reported have a biological rationale such as *ANKRD9*, which is involved in lipid metabolism and has the highest sequence similarity to phospholipase A_2_, an enzyme for the release of fatty acid from phospholipids (*35*). Hispanic populations are underrepresented in current metQTL-associations databases, and differences in some metQTL-associations between ethnicities have been reported (*6*), which may also explain why some GACRS findings did not replicate in CAMP.

**Table 4.** Overlapping metQTL-associations (*n* = 47) across models, which were confirmed by permutation-based testing in each model. Associations were investigated for previous reporting in the literature based on the GWAS catalog (www.ebi.ac.uk/gwas/home) (last column). DMGV, Dimethylguanidino valeric acid; GABA, γ-aminobutyric acid; LPC, lysophosphatidylcholine; LPE, Lysophosphatidylethanolamine; PC, phosphatidylcholine; PE, phosphatidylethanolamine; PS, Phosphatidylserine; SDMA, Symmetric dimethylarginine; SM, Sphingomyelin.

Metabolite	Gene	ID	A1	GACRS with model 2				CAMP with model 2			GWAS catalog
				MAF	β	SE	*P*	β	SE	*P*	Reported SNP/gene; similar metabolite if the metabolite is different
1-Methyladenosine	*ADA*	rs406383^*^	G	0.309	−0.447	0.044	7.85E−23	−0.326	0.065	6.03E−07	rs406383/*ADA*
2-Aminoisobutyric acid	*AGXT2*	rs344506^*^	T	0.029	−1.023	0.122	1.86E−16	−0.767	0.185	3.74E−05	rs638662/*AGXT2*
2-Aminoisobutyric acid	*AGXT2*	rs37376^*^	G	0.219	0.888	0.047	3.86E−69	0.831	0.076	5.86E−26	rs37370/*AGXT2*
2-Aminooctanoate	*STAMBP*	rs11893881	A	0.305	−0.559	0.044	1.28E−34	–	–	–	rs7603647/*STAMBP*
Acetylgalactosamine	*C4orf33*	rs1709422	C	0.376	0.366	0.044	6.19E−16	–	–	–	rs1709422/*C4orf33*
Acisoga	*RNF167*	rs12951677^*^	T	0.489	−0.304	0.042	1.19E−12	−0.154	0.055	4.89E−03	rs10445262/*RNF167*
Arginine	*ARG1*	rs17788484^*^	T	0.045	−0.740	0.109	1.62E−11	−0.701	0.146	2.03E−06	rs17788484/*ARG1*
Betaine	*DMGDH*	rs2461249^*^	A	0.447	−0.317	0.043	4.92E−13	−0.214	0.053	6.75E−05	rs2431951/*DMGDH*
Biliverdin	*UGT1A6*	rs1976391^*^	G	0.289	0.637	0.044	3.36E−43	0.692	0.049	1.27E−40	rs1976391/*UGT1A6*
C20:4 carnitine	*SLC22A16*	rs12210538^*^	G	0.171	−0.488	0.056	8.44E−18	−0.278	0.063	1.31E−05	rs12210538/*SLC22A16*; tetradecanoyl-carnitine
C26 carnitine	*TESMIN*	rs11826602^*^	G	0.127	0.432	0.063	1.50E−11	0.380	0.076	6.18E−07	–
C3-DC-CH3 carnitine	*LACTB*	rs2729835^*^	A	0.486	−0.321	0.043	2.25E−13	−0.396	0.055	2.11E−12	rs4775623/*LACTB*
C4 carnitine	*ACADS*	rs1799958^*^	A	0.330	0.589	0.043	3.65E−39	0.611	0.058	2.00E−24	rs1799958/*ACADS*
C5-DC carnitine	*CPT2*	rs2229291^*^	G	0.051	−0.718	0.097	2.85E−13	−0.574	0.276	3.78E−02	–
Carnitine	*SLC16A9*	rs1171614^*^	T	0.169	−0.454	0.057	4.49E−15	−0.489	0.059	4.27E−16	rs1171614/*SLC16A9*
Choline	*SPATA45*	rs6666277	G	0.375	−0.317	0.046	6.10E−12	−0.038	0.051	4.56E−01	rs2047287/*SPATA45*
DMGV	*SLC45A2*	rs250417^*^	G	0.290	−0.362	0.044	3.73E−16	−0.303	0.083	2.94E−04	–
DMGV	*AGXT2*	rs37371^*^	A	0.218	−0.828	0.043	9.35E−71	−0.702	0.072	5.49E−21	rs37369/*AGXT2*
GABA	*SLC1A4*	rs2160387^*^	T	0.490	−0.333	0.043	1.85E−14	−0.261	0.053	9.99E−07	rs2160387/*SLC1A4*
Linoleoyl ethanolamide	*FAAH*	rs1571138^*^	A	0.274	0.359	0.047	7.33E−14	0.259	0.060	2.01E−05	rs1571138/*FAAH*
LPC(20:4)	*FADS2*	rs1535^*^	G	0.483	−0.520	0.041	1.43E−34	−0.418	0.053	8.25E−15	rs1535/*FADS2*
LPC(22:4)	*FADS1*	rs174566^*^	A	0.495	0.318	0.042	9.47E−14	0.156	0.054	4.07E−03	rs174566/*FADS1*; LPC(20:4)
LPC(22:5)	*FADS2*	rs174580^*^	A	0.483	0.364	0.042	2.34E−17	0.188	0.053	4.82E−04	rs174580/*FADS2*; LPC(20:4)
LPE(20:4)	*FADS2*	rs1535^*^	G	0.483	−0.343	0.043	5.21E−15	−0.189	0.056	8.82E−04	rs1535/*FADS2*
*N*_6_,*N*_6_-dimethyllysine	*PIK3AP1*	rs563654^*^	T	0.300	−0.348	0.048	7.63E−13	−0.211	0.080	8.69E−03	–
*N*_6_,*N*_6_-dimethyllysine	*PYROXD2*	rs2147896^*^	A	0.440	0.937	0.033	6.27E−131	0.969	0.035	6.15E−113	rs2147896/*PYROXD2*
*N*_6_,*N*_6_-dimethyllysine	*HPSE2*	rs4423123	G	0.486	−0.299	0.043	1.02E−11	−0.036	0.049	4.62E−01	–
*N*_6_-acetyllysine	*STAMBP*	rs28879089^*^	A	0.290	0.352	0.047	2.02E−13	0.343	0.061	2.06E−08	rs7594511/*STAMBP*
*N*_6_-methyllysine	*PIK3AP1*	rs12217499^*^	A	0.239	−0.371	0.052	2.79E−12	−0.157	0.087	6.97E−02	–
*N*_6_-methyllysine	*PYROXD2*	rs2147896^*^	A	0.440	1.088	0.028	2.62E−199	1.018	0.032	2.07E−137	rs2147896/*PYROXD2*
*N*_6_-methyllysine	*HPSE2*	rs4423123	G	0.486	−0.327	0.043	9.94E−14	−0.010	0.049	8.42E−01	–
*N*-acetylornithine	*STAMBP*	rs10168931^*^	A	0.295	−0.827	0.040	6.56E−81	−0.853	0.052	1.48E−50	rs10168931/*STAMBP*
*N*-α-acetylarginine	*STAMBP*	rs7596773^*^	T	0.295	0.997	0.037	7.90E−122	0.924	0.052	3.34E−59	rs7596773/*STAMBP*
PC(P-36:4)/PC(O-36:5)	*FADS2*	rs174600	C	0.472	−0.293	0.043	1.15E−11	−0.087	0.055	1.14E−01	rs174564/*FADS2*
PE(34:2)	*LIPC*	rs2070895^*^	A	0.439	0.301	0.045	3.54E−11	0.309	0.061	4.58E−07	rs2070895/*LIPC*
PE(36:4)	*LIPC*	rs2070895^*^	A	0.439	0.301	0.045	4.57E−11	0.318	0.061	2.38E−07	rs2070895/*LIPC*
PE(38:6)	*ALDH1A2*	rs35853021^*^	T	0.341	0.387	0.045	5.82E−17	0.281	0.056	5.75E−07	rs35853021/*ALDH1A2*
PE(38:6)	*LIPC*	rs2070895^*^	A	0.439	0.523	0.042	1.37E−32	0.364	0.060	1.67E−09	rs2070895/*LIPC*; PE(38:5)
PE(40:6)	*ALDH1A2*	rs35853021^*^	T	0.341	0.318	0.044	6.03E−13	0.200	0.056	3.71E−04	rs35853021/*ALDH1A2*; PE(38:6)
PE(40:6)	*LIPC*	rs2070895^*^	A	0.439	0.399	0.041	3.75E−21	0.267	0.060	1.06E−05	rs2070895/*LIPC*; PE(40:5)
PS(P-36:2)/PS(O-36:3)	*HEATR4*	rs1074501^*^	C	0.230	0.581	0.048	2.54E−31	0.417	0.058	1.34E−12	–
SDMA	*PYROXD2*	rs2147896^*^	A	0.440	0.583	0.041	1.26E−42	0.796	0.043	7.03E−63	rs2147896/*PYROXD2*
SM(d18:1/14:0)	*PPP2R5E*	rs7160525^*^	A	0.262	0.373	0.047	9.76E−15	0.405	0.064	4.63E−10	rs7160525/*PPP2R5E*
SM(d18:1/20:0)	*PPP2R5E*	rs7160525^*^	A	0.262	0.349	0.048	5.04E−13	0.361	0.065	3.43E−08	rs7160525/*PPP2R5E*
Urate	*SLC2A9*	rs7696983^*^	A	0.300	−0.460	0.045	1.87E−23	−0.471	0.054	1.46E−17	rs7442295/*SLC2A9*
Xanthosine	*GMPR*	rs6933491^*^	G	0.495	0.289	0.043	4.46E−11	0.166	0.055	2.51E−03	rs9477074/*GMPR*
Xanthosine	*ANKRD9*	rs1190550	T	0.351	0.301	0.044	1.28E−11	0.035	0.061	5.66E−01	–

*Replicated metQTL-association, which is defined as an association *P* < 5% in CAMP with a consistent direction of effect.

## DISCUSSION

There is an increasing number of mGWASs and metQTL studies in the recent literature (*2*, *3*, *5*, *7*–*11*). However, while several specific methodological approaches and tools are available for the analysis of other omics layers, such as gene expression or DNA methylation, the genetic analysis of metabolite data is currently routinely performed using standard GWAS methodology. At the same time, the nature of metabolite data with skewed data distributions and outlier values is generally recognized, and often, data transformations are applied before analysis to meet the distributional assumptions of the established association testing approaches. However, no gold standard exists for the genetic analysis of metabolite data, and analysis guidelines are missing. Up to now, log transformation in mGWASs has been popularly adopted, and a substantial number of reported metQTLs in these analyses were low-frequency or rare variants (*3*, *9*, *10*).

Here, we explore the performance of different mGWAS/metQTL analysis approaches using WGS and metabolite data for two family-based studies of childhood asthma. Our investigations incorporate different data transformations, population-based and family-based analysis approaches, permutation-based testing, and robust Huber regression. The findings and conclusions derived from the real data analysis are supported by two simulation studies.

The population-based analysis based on unrelated children with asthma using log transformation resulted in a substantially larger number of detected metQTLs compared to the analysis using the rank inverse normal transformation. However, careful disentanglement of these metQTL-associations revealed that low-frequency and rare genetic variations in combination with residual data distribution skewness were the main drivers of what we determined to be false-positive associations, which was supported by the observations in the first simulation study. Permutation-based analysis and Huber regression also confirmed these observations. The low replication rate in the CAMP replication analysis provided additional evidence for this claim.

The metQTL-associations identified on the basis of the rank inverse normal transformation had a significantly higher replication rate in the CAMP data and were largely supported by permutation-based testing and Huber regression. The fully adjusted two-step approach detected a large number of metQTL-associations that involved only two metabolites. Further investigation showed that these two metabolites have strong outlier values and that the first step regression based on untransformed metabolite levels introduces outlier values in the second step regression based on rank inverse normal–transformed residuals. This can lead to false-positive findings when analyzing rare and low-frequency genetic variants, which were illustrated in the second simulation study. We note that the two-step approach provided more reliable results in our analysis when we performed the log transformation first (Fig. 3F). A recent study on the WGS for human metabolome in the National Heart, Lung, and Blood Institute (NHLBI) Trans-Omics for Precision Medicine (TOPMed) cohorts used a two-step approach with two rank inverse normal transformations where most of the novel metQTL-associations were low-frequency variants under 5% (*6*). Moreover, low-frequency or rare variants have been highlighted with a larger effect on metabolite levels compared to common variants (*6*, *7*). Therefore, the issue that we observed should be further examined for low-frequency variants reported in metQTL-associations to ensure reproducible results.

The family-based analysis provided similar results for both the log transformation and the rank inverse normal transformation. Overall, family-based analysis using the FBAT approach was less susceptible to false-positive associations, which is suggested to be a result of the effectively smaller sample size and the robust test statistic. FBAT’s nonparametric conditioning nature and insensitivity regarding phenotype distributional assumptions make it robust to skewed data and population stratification. However, in general, these strengths come at the cost of reduced power. We did not observe evidence that potential false-positive findings are linked to insufficient adjustment for population stratification in the population-based analyses.

The strengths of our analysis include the discovery and replication analysis approach in two family-based studies of childhood asthma with consistent data generation procedures and a successful history of replicating results (*36*–*38*). Because both studies consist of children with a trio-based structure, we are in a position to compare population-based and family-based analysis approaches. Furthermore, the observations and findings from the analysis of these two studies are supported by simulation studies that illustrate the underlying technical challenges of genetic metabolite data analysis. The inclusion of multiple metabolites allowed us to evaluate the robustness of methods across a range of metabolite distributions and characteristics.

Nevertheless, our investigations have several limitations. First, both studies are ascertained based on offspring asthma status. Therefore, the genetic and metabolomic data may be affected by the characteristics of this study population. Second, the sample size in our analysis is small compared to recent metQTL studies or mGWASs. Moreover, the metabolomic data here concentrate on the untargeted liquid chromatography–mass spectrometry (LC-MS) platform only, and other metabolite platforms might have different data distribution features. However, similar results were found across different platforms such as C8 and C18 columns. Furthermore, the small sample size in the CAMP replication analysis is a limiting factor, particularly for low-frequency variants. We thus note that a lack of replication in CAMP does not necessarily indicate false positives in the discovery cohort. Third, our study focused on mGWASs and the corresponding statistical characteristics for each metabolite separately and did not investigate pleiotropy, which could reveal shared genetic associations across metabolites. Fourth, we did not consider all possible analysis approaches including quantile regression, which could be a valuable and distribution-agnostic approach. In addition, a fully nonparametric version, which does not assume an additive effect for SNPs (number of minor alleles coding), is an interesting direction for future research. Fifth, although we highlight the connection between data distribution skewness and false-positive findings, we are not in the position yet to provide an explicit cutoff value for a skewness metric to guide analysis based on a general rule. Last, despite the mentioned increased computational burden of Huber regression and especially permutation-based testing, we did not perform runtime comparisons due to systematic differences in the efficiency of the underlying platforms and implementations. We emphasize again that Huber regression and permutation-based testing could be considered as additional analysis approaches for validation, applied to selected significant associations.

In conclusion, our findings demonstrate that the genetic analysis of metabolite data requires careful consideration. Log transformations generally reduce the skewness of data distributions, but especially in the analysis of rare and low-frequency genetic variants, false-positive results can still arise. Notably, we also observed metabolites for which log transformation increases the type 1 error rate compared to untransformed metabolite levels. The rank inverse normal transformation provides a robust and computationally feasible approach that was able to control type 1 error rates in all our considerations. However, the application of data transformations can potentially lead to false negatives when the characteristics of the data are substantially altered. Here, the permutation-based approach and Huber regression can be valuable alternatives, although they are computationally expensive and require nonstandard analysis tools. We note again that the two-step approach showed more robustness in our simulations when we applied a log transformation first. Future mGWAS analyses should weigh up the strengths and drawbacks of these approaches and determine which is most well suited for their specific research question and context.

## MATERIALS AND METHODS

### Discovery and replication populations

GACRS is a study of children with asthma and their parents (affected offspring trios). In addition to having at least two respiratory symptoms (wheezing, coughing, or dyspnea) or a history of asthma episodes in the previous year, all participants were aged 6 to 14 and had been diagnosed with asthma by a physician (*28*). To ensure that they were descended from an original population that was primarily made up of Spanish and Amerindians, all participants also had at least six great-grandparents who were born in Costa Rica’s Central Valley. CAMP is a multiethnic cohort of North American children aged 5 to 12 at baseline with mild to moderate asthma [symptoms for >6 months in the year before interview and provocative concentration of methacholine causing a 20% drop (PC20) in Forced expiratory volume in one second (FEV1) < 12.5 mg/ml] (*26*). CAMP was originally designed as a multicenter clinical trial to determine the long-term effects of three inhaled treatments for childhood asthma (*26*).

### WGS data

WGS data for GACRS and CAMP were generated as part of the TOPMed Program (*39*). Details regarding the laboratory methods, data processing, and quality control (QC; freeze 10 release) are described on the TOPMed website and in documents included in the TOPMed accession release in the database of genotypes (accession numbers: phs000988 and phs001726, respectively). Relatedness and pedigree structures were investigated and confirmed using genome-wide identity-by-descent estimates generated by KING (Kinship-based INference for Gwas) (*40*). Sex mismatches were identified using PLINK2 (*41*). We considered only biallelic autosomal SNPs for our analyses. SNPs with genotyping rate (<98%), MAF (<1%), or deviations from Hardy-Weinberg proportions (*P* < 10^−8^) were removed.

### Metabolomic profiling using the HILIC-positive platform

In both studies, metabolomic plasma profiling was generated as part of the TOPMed initiative (*29*). Nontargeted LC-MS methods using high-resolution, accurate mass profiling were conducted at the Broad Institute. Metabolites were identified by mass/charge ratio, by retention time, and through a comparison to a library of purified known standards. Data were processed according to the standard QC pipelines, which were previously provided in detail (*29*). In brief, pooled plasma QC samples were included throughout the assay after intervals of approximately 20 study samples. Metabolites with the coefficient of variation (CV%) > 25% or missing > 75% were excluded. The remaining missing values were imputed using the *k*-nearest neighbor imputation method (R package “VIM”) (*42*). Metabolites were matched between GACRS and CAMP, and only metabolites that passed QC in both studies were included in the analysis. For the purposes of our method comparison analyses, we focus only on a single profiling platform, HILIC-positive, which measures polar metabolites, including amino acids, acylcarnitines, and amines (*n* = 195 metabolites). The skewness of each metabolite was calculated with the “skewness()” function from the “e1071” R package (*43*).

### Calculation of PCs of genetic ancestry

The PCs of genetic ancestry for the population-based analyses were generated using the variance-standardized relationship matrix in PLINK2 based on an linkage disequilibrium (LD)-pruned set of SNPs (window size of 50 kb, step size of 5 markers, and pairwise *r*^2^ < 0.5).

### Statistical analysis

#### Population-based approach based on unrelated offspring data

Associations between SNPs and metabolite levels in unrelated offspring were tested using different linear regression models. In model 1, the metabolite levels were log_10_ transformed and Pareto scaled after imputation of missing values. Pareto scaling subtracts the mean and divides by the square root of the SD, and is a commonly used technique in the analysis of metabolomic data (*14*). We note that for linear regression analyses, this scaling does not change the association results.

Age, sex, BMI, and the top 10 PCs of genetic ancestry were included as covariates. Model 2 incorporated the same set of covariates, but the metabolite levels were rank inverse normal transformed before the regression analysis. A Pareto scaling before the rank inverse normal transformation is not necessary here because the transformation uses only the ranks that are preserved by the Pareto scaling. In the third model, model 3, a fully adjusted two-step approach was used (*30*): Step 1: Untransformed metabolite levels were regressed on the same set of covariates as in models 1 and 2. Step 2: The corresponding residuals were rank inverse normal transformed and incorporated in a second step regression analysis using the same covariates (*7*, *30*). The reported *P* value corresponds to the coefficient of the SNP in the second step regression. All analyses used metabolite level as the outcome and assumed an additive genetic model.

#### Family-based approach based on trio data

We applied family-based association approaches as a comparison. For the family-based analysis using FBAT (*31*), we specified the outcome variable as the residuals after regressing transformed metabolite levels on age, sex, and BMI. FBAT does not require population structure adjustments, as it is robust to genetic stratification (*25*). Thus, no adjustment for PCs of genetic ancestry was applied for the family-based approaches. We considered two different data transformations: log_10_ transformation (model 4) and rank inverse normal transformation (model 5). The two-step approach was not applied for the family-based analysis because FBAT is a conditional score test and does not fit a model. All FBAT analyses assumed an additive genetic model.

#### Identifying independently significant SNP-metabolite associations

The significance of the association between an SNP and a metabolite in a given model was determined by a threshold *P* value of less than 5 × 10^−8^/195 ≒ 2.56 × 10^−10^ (standard genome-wide significance level adjusted for the number of metabolites using a Bonferroni correction). To obtain independent associations, LD clumping was applied using PLINK2 and the following parameters: --clump-r2 0.001 & --clump-kb 1000 (*r*^2^ < 0.1% at least 1 Mb apart).

#### Permutation-based tests and Huber regression

We further applied permutation-based testing and Huber regression to evaluate the reliability of the observed SNP-metabolite associations in GACRS. This analysis was performed for the Bonferroni significant findings based on the population-based models 1 to 3 in GACRS. The rationale is that outlier values and skewness of the metabolite data distributions can lead to inaccuracies of the asymptotic *P* values in these models. Permutation-based testing and Huber regression are more robust approaches that aim to maintain valid type 1 error rates in more broad scenarios.

Permutation-based testing has the advantage that it requires weaker assumptions about the data distribution. PLINK2 performs permutation-based testing by randomly permuting the phenotype data and recomputing association test statistics to estimate empirical *P* values. The limitation here is the computational burden, limiting the number of permutations by 1 × 10^9^ [technical maximum in the PLINK2 software (*41*)] and therefore restricting the results of permutation-based testing to a minimum permutation *P* value of 1 × 10^−9^. We considered an empirical *P* value of <1 × 10^−9^ as additional evidence for the metQTLs representing a true signal and not a statistical artifact.

The same significant SNP-metabolite associations from models 1 to 3 were additionally analyzed using robust regression (Huber regression). Huber regression was designed to be less sensitive to outliers in the data compared to the ordinary linear regression used in models 1 to 3 (*20*). We investigated the consistency of findings, in terms of how many regression hits replicated with permutation or the Huber regression, and in terms of how many of the permutation/Huber hits were among the metQTL-associations in models 1 to 3.

#### Validation in an independent population

For models 1 to 5 in GACRS, we sought to replicate the significant findings in CAMP, based on a *P* value of <0.05 with a concordant direction of effect as the replication criteria.

### Consent statement

Written parental consent and participating child’s assent were obtained. For GACRS data, the study was approved by the Mass General Brigham Human Research Committee at Brigham and Women’s Hospital (Boston, MA; protocol no. 2000P001130) and the Hospital Nacional de Niños (San José, Costa Rica). For CAMP data, all study procedures were approved by the Mass General Brigham Human Research Committee at Brigham and Women’s Hospital (Boston, MA; protocol no. 2011P000710).

## Supplementary Materials

### The PDF file includes:

Fig. S1 Data S1. Legends of Tables S1 to S5 Data S2. NHLBI Trans-Omics for Precision Medicine (TOPMed) Consortium
