# Supplementary material for: On the analysis of metabolite quantitative trait loci: Impact of different data transformations and study designs
Source: Sci Adv. 2025 Apr 11;11(15):eadp4532. doi: 10.1126/sciadv.adp4532 (PMC11988406; doi:10.1126/sciadv.adp4532)
Supplement: Supplementary file 1 — Fig. S1 Data S1. Legends of Tables S1 to S5 Data S2. NHLBI Trans-Omics for Precision Medicine (TOPMed) Consortium [file sciadv.adp4532_sm.pdf]

Science Advances
AAAS

Supplementary Materials for

On the analysis of metabolite quantitative trait loci: Impact of different data transformations and study designs

Sanghun Lee *et al.*

Corresponding author: Jessica A. Lasky-Su, rejas@channing.harvard.edu;
Julian Hecker, rejhe@channing.harvard.edu

Sci. Adv. **11**, eadp4532 (2025)
DOI: 10.1126/sciadv.adp4532

The PDF file includes:

Fig. S1
Data S1. Legends of Tables S1 to S5
Data S2. NHLBI Trans-Omics for Precision Medicine (TOPMed) Consortium

Other Supplementary Material for this manuscript includes the following:

Data S1

Fig. S1.

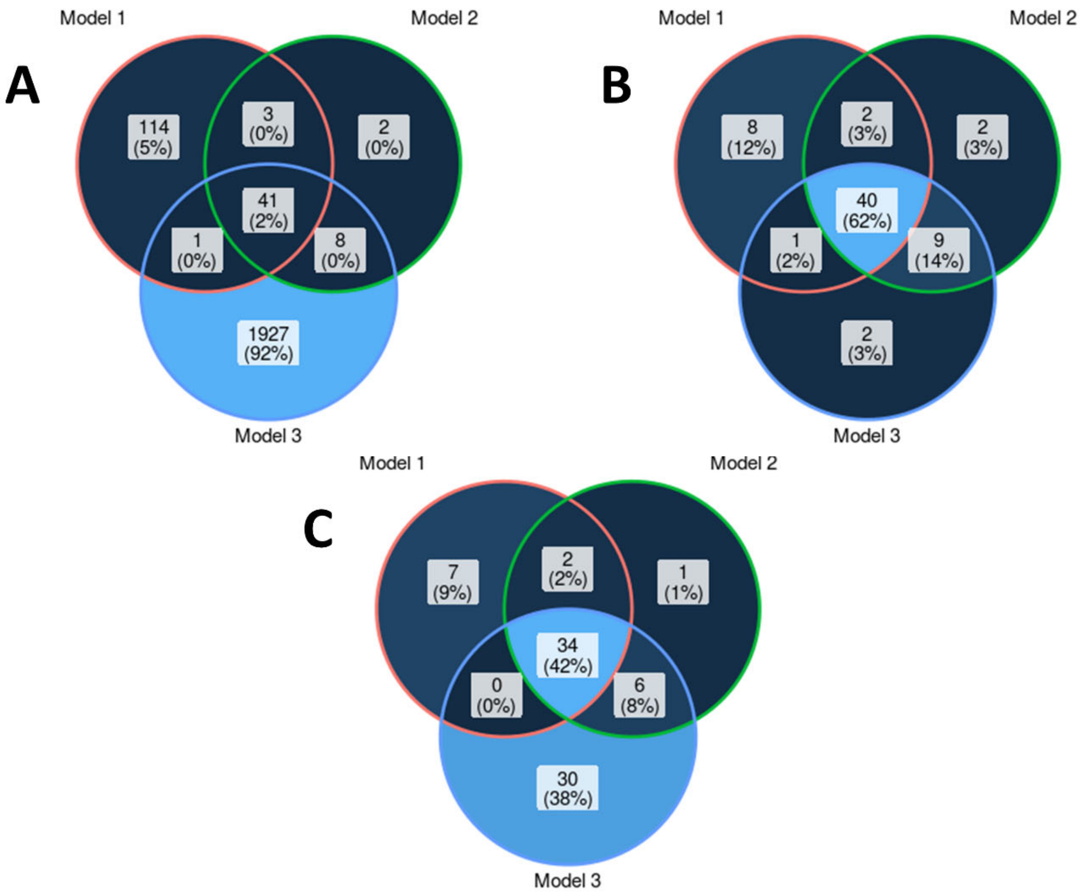

(A) Venn diagram for the number of metQTL-associations identified in Models 1-3 in GACRS. (B) Venn diagram for the number of metQTL-associations confirmed by permutation-based tests in Models 1-3. (C) Venn diagram for the number of metQTL-associations replicated in CAMP in Models 1-3.

Data S1. Contains tables S1 to S5. (Provided as a separate file in Excel.)

Table S1-S3. The significant metQTL associations based on the permutation-based tests of Model 1, 2, and 3, where the results in each Model are compared.

Table S4. The skewness of metabolites using untransformed values, log10-transformed values, and rank inverse normal transformed values, and summary results of the first simulation study (n=1,000).

For each of the 195 metabolites, the number of false positive findings for untransformed metabolite levels, log10-transformed data, and rank inverse normal transformed data among 10,000 simulations using genotypes for a minor allele frequency of 1% is reported. False positives are identified based on a Bonferroni correction for 10,000 replicates and a significance level of 0.05. Additionally, the corresponding skewness of the respective data distributions is shown.

Table S5. Metabolite-specific results of the first simulation study with the sample sizes (n=1,000, 5,000, and 10,000)

Data S2. NHLBI Trans-Omics for Precision Medicine (TOPMed) Consortium

A list of all NHLBI Trans-Omics for Precision Medicine (TOPMed) Consortium authors and collaborators and their affiliations

Name	Institution(s)	Primary Department	Project Group	Sponsoring Pi	Institution City	Institution State	Zip Code	Country
Abe, Namiko	New York Genome Center		New York Genome Center Genomics	Soren Germer	New York	New York	10013	United States of America
Abecasis, Gonçalo	University of Michigan		IRC	Gonçalo Abecasis	Ann Arbor	Michigan	48109	United States of America
Aguet, Francois	Broad Institute		Broad Institute Genomics Platform	Stacey Gabriel	Cambridge	Massachusetts	2142	United States of America
Albert, Christine	Cedars Sinai		AFGen	Patrick Ellinor	Boston	Massachusetts	2114	United States of America
Almasy, Laura	Children's Hospital of Philadelphia, University of Pennsylvania		SAFS	John Blangero	Philadelphia	Pennsylvania	19104	United States of America
Alonso, Alvaro	Emory University		AFGen, VTE	Eric Boerwinkle	Atlanta	Georgia	30322	United States of America
Alonso, Alvaro	Emory University		AFGen, VTE	Patrick Ellinor	Atlanta	Georgia	30322	United States of America
Ament, Seth	University of Maryland		Amish	Braxton D. Mitchell	Baltimore	Maryland	21201	United States of America
Anderson, Peter	University of Washington		Northwest Genomics Center	Evan Eichler	Seattle	Washington	98195	United States of America
Anugu, Pramod	University of Mississippi		JHS	Laura Raffield	Jackson	Mississippi	38677	United States of America
Ardlie, Kristin	Broad Institute		Broad Institute Genomics Platform	Stacey Gabriel	Cambridge	Massachusetts	2142	United States of America
Arking, Dan	Johns Hopkins University		AFGen, Analysis Program, MESA, VTE	Patrick Ellinor	Baltimore	Maryland	21218	United States of America
Arking, Dan	Johns Hopkins University		AFGen, Analysis Program, MESA, VTE	Jerome Rotter	Baltimore	Maryland	21218	United States of America
Arking, Dan	Johns Hopkins University		AFGen, Analysis Program, MESA, VTE	Eric Boerwinkle	Baltimore	Maryland	21218	United States of America
Arnett, Donna K	University of South Carolina	Executive VP of Academic Affairs and Provost	GOLDN, HyperGEN_GENOA	Donna K Arnett	Columbia	South Carolina	29208	United States of America
Arnett, Donna K	University of South Carolina	Executive VP of Academic Affairs and Provost	GOLDN, HyperGEN_GENOA	Donna K Arnett	Columbia	South Carolina	29208	United States of America
Ashley-Koch, Allison	Duke University		OMG_SCD	Allison Ashley-Koch	Durham	North Carolina	27708	United States of America
Aslibekyan, Stella	University of Alabama		GOLDN	Donna K Arnett	Birmingham	Alabama	35487	United States of America
Assimes, Tim	Stanford University		WHI	Charles Kooperberg	Stanford	California	94305	United States of America
Auer, Paul	Medical College of Wisconsin		JHS, WHI	Charles Kooperberg	Milwaukee	Wisconsin	53211	United States of America
Auer, Paul	Medical College of Wisconsin		JHS, WHI	Laura Raffield	Milwaukee	Wisconsin	53211	United States of America
Avramopoulos, Dimitrios	Johns Hopkins University		SIT_SCD	James Casella	Baltimore	Maryland	21218	United States of America
Ayas, Najib	Providence Health Care	Medicine	GEM-OSA	Allan Pack	Vancouver			Canada
Balasubramanian, Adithya	Baylor College of Medicine Human Genome Sequencing Center		Baylor	Richard Gibbs	Houston	Texas	77030	United States of America
Barnard, John	Cleveland Clinic		AFGen, PVDOMICS	Patrick Ellinor	Cleveland	Ohio	44195	United States of America

Barnard, John	Cleveland Clinic		AFGen, PVDOMICS	Serpil Erzurum	Cleveland	Ohio	44195	United States of America
Barnes, Kathleen	Tempus, University of Colorado Anschutz Medical Campus	Medicine	BAGS	Kathleen Barnes	Aurora	Colorado	80045	United States of America
Barr, R. Graham	Columbia University		Analysis Program, COPD, MESA	Ed Silverman	New York	New York	10032	United States of America
Barr, R. Graham	Columbia University		Analysis Program, COPD, MESA	Jerome Rotter	New York	New York	10032	United States of America
Barron-Casella, Emily	Johns Hopkins University		SIT_SCD	James Casella	Baltimore	Maryland	21218	United States of America
Barwick, Lucas	The Emmes Corporation	LTRC	LTRC	Ed Silverman	Rockville	Maryland	20850	United States of America
Beaty, Terri	Johns Hopkins University		BAGS, COPD	Ed Silverman	Baltimore	Maryland	21218	United States of America
Beaty, Terri	Johns Hopkins University		BAGS, COPD	Kathleen Barnes	Baltimore	Maryland	21218	United States of America
Beck, Gerald	Cleveland Clinic	Quantitative Health Sciences	PVDOMICS	Serpil Erzurum	Cleveland	Ohio	44195	United States of America
Becker, Diane	Johns Hopkins University	Medicine	GeneSTAR	Lisa Yanek	Baltimore	Maryland	21218	United States of America
Becker, Lewis	Johns Hopkins University		GeneSTAR	Lisa Yanek	Baltimore	Maryland	21218	United States of America
Beitelshees, Amber	University of Maryland		Amish	Braxton D. Mitchell	Baltimore	Maryland	21201	United States of America
Benos, Takis	University of Pittsburgh		Analysis Program, COPD	Ed Silverman	Pittsburgh	Pennsylvania	15260	United States of America
Bezerra, Marcos	Funda 챌찻 o de Hematologia e Hemoterapia de Pernambuco - Hemope		Boston-Brazil_SCD	Vijay G. Sankaran	Recife		52011-000	Brazil
Bielak, Larry	University of Michigan		AA_CAC, HyperGEN_GENOA	Donna K Arnett	Ann Arbor	Michigan	48109	United States of America
Bielak, Larry	University of Michigan		AA_CAC, HyperGEN_GENOA	Kent D. Taylor	Ann Arbor	Michigan	48109	United States of America
Bis, Joshua	University of Washington	Cardiovascular Health Research Unit, Department of Medicine	CHS	Bruce Psaty	Seattle	Washington	98195	United States of America
Bis, Joshua	University of Washington	Cardiovascular Health Research Unit, Department of Medicine	CHS		Seattle	Washington	98195	United States of America
Blangero, John	University of Texas Rio Grande Valley School of Medicine	Human Genetics	SAFS	John Blangero	Brownsville	Texas	78520	United States of America
Blue, Nathan	University of Utah	Obstetrics and Gynecology	nuMoM2b-HHS	Nathan Blue	Salt Lake City	Utah	84132	United States of America
Bowden, Donald W.	Wake Forest Baptist Health	Department of Biochemistry	AA_CAC	Kent D. Taylor	Winston-Salem	North Carolina	27157	United States of America
Bowler, Russell	Cleveland Clinic	National Jewish Health	COPD, COPDMet	Russell Bowler	Denver	Colorado	80206	United States of America
Bowler, Russell	Cleveland Clinic	National Jewish Health	COPD, COPDMet	Ed Silverman	Denver	Colorado	80206	United States of America
Brody, Jennifer	University of Washington		CHS	Bruce Psaty	Seattle	Washington	98195	United States of America

Brody, Jennifer	University of Washington		CHS		Seattle	Washington	98195	United States of America
Broeckel, Ulrich	Medical College of Wisconsin	Pediatrics	HyperGEN_GENOA	Donna K Arnett	Milwaukee	Wisconsin	53226	United States of America
Broome, Jai	University of Washington				Seattle	Washington	98195	United States of America
Brown, Deborah	University of Texas Health at Houston	Pediatrics	HIPS	Deborah Brown	Houston	Texas	77030	United States of America
Bunting, Karen	New York Genome Center		New York Genome Center Genomics	Soren Germer	New York	New York	10013	United States of America
Burchard, Esteban	University of California, San Francisco		PGX_Asthma	Esteban Burchard	San Francisco	California	94143	United States of America
Bustamante, Carlos	Stanford University	Biomedical Data Science	SCVI	Joseph Wu	Stanford	California	94305	United States of America
Buth, Erin	University of Washington	Biostatistics			Seattle	Washington	98195	United States of America
Cade, Brian	Brigham & Women's Hospital	Brigham and Women's Hospital	CFS, Samoan	Susan Redline	Boston	Massachusetts	2115	United States of America
Cade, Brian	Brigham & Women's Hospital	Brigham and Women's Hospital	CFS, Samoan	Ryan L Minster	Boston	Massachusetts	2115	United States of America
Cardwell, Jonathan	University of Colorado at Denver		IPF	David Schwartz	Denver	Colorado	80204	United States of America
Carey, Vincent	Brigham & Women's Hospital		ECLIPSE	Ed Silverman	Boston	Massachusetts	2115	United States of America
Carrier, Julie	University of Montreal		GEM-OSA	Allan Pack				United States of America
Carson, April P.	University of Mississippi	Medicine	JHS	Laura Raffield	Jackson	Mississippi	39213	United States of America
Carty, Cara	Washington State University		WHI	Charles Kooperberg	Pullman	Washington	99164	United States of America
Casaburi, Richard	University of California, Los Angeles		COPD	Ed Silverman	Los Angeles	California	90095	United States of America
Casas Romero, Juan P	Brigham & Women's Hospital		BCC-PREG	Kathryn J. Gray				United States of America
Casella, James	Johns Hopkins University		SIT_SCD	James Casella	Baltimore	Maryland	21218	United States of America
Castaldi, Peter	Brigham & Women's Hospital	Medicine	COPD, ECLIPSE, LTRC	Ed Silverman	Boston	Massachusetts	2115	United States of America
Castaldi, Peter	Brigham & Women's Hospital	Medicine	COPD, ECLIPSE, LTRC	Ed Silverman	Boston	Massachusetts	2115	United States of America
Castaldi, Peter	Brigham & Women's Hospital	Medicine	COPD, ECLIPSE, LTRC	Ed Silverman	Boston	Massachusetts	2115	United States of America
Chaffin, Mark	Broad Institute		AFGen	Patrick Ellinor	Cambridge	Massachusetts	2142	United States of America
Chang, Christy	University of Maryland		Amish	Braxton D. Mitchell	Baltimore	Maryland	21201	United States of America
Chang, Yi-Cheng	National Taiwan University		THRV	Yii-Der Ida Chen	Taipei		10617	Taiwan (Province of China)
Chasman, Daniel	Brigham & Women's Hospital	Division of Preventive Medicine	AFGen	Patrick Ellinor	Boston	Massachusetts	2215	United States of America
Chavan, Sameer	University of Colorado at Denver		BAGS	Kathleen Barnes	Denver	Colorado	80204	United States of America
Chen, Bo-Juen	New York Genome Center		New York Genome Center Genomics	Soren Germer	New York	New York	10013	United States of America
Chen, Wei-Min	University of Virginia		MESA	Jerome Rotter	Charlottesville	Virginia	22903	United States of America

Chen, Yii-Der Ida	Lundquist Institute		AA_CAC, MESA, THRV	Kent D. Taylor	Torrance	California	90502	United States of America
Chen, Yii-Der Ida	Lundquist Institute		AA_CAC, MESA, THRV	Jerome Rotter	Torrance	California	90502	United States of America
Chen, Yii-Der Ida	Lundquist Institute		AA_CAC, MESA, THRV	Yii-Der Ida Chen	Torrance	California	90502	United States of America
Choi, Seung Hoan	Broad Institute		AFGen, Analysis Program	Patrick Ellinor	Cambridge	Massachusetts	2142	United States of America
Chuang, Lee-Ming	National Taiwan University	National Taiwan University Hospital	MESA, THRV	Jerome Rotter	Taipei		10617	Taiwan (Province of China)
Chuang, Lee-Ming	National Taiwan University	National Taiwan University Hospital	MESA, THRV	Yii-Der Ida Chen	Taipei		10617	Taiwan (Province of China)
Chung, Mina	Cleveland Clinic	Cleveland Clinic	AFGen	Patrick Ellinor	Cleveland	Ohio	44195	United States of America
Chung, Ren-Hua	National Health Research Institute Taiwan		THRV	Yii-Der Ida Chen	Miaoli County		350	Taiwan (Province of China)
Clish, Clary	Broad Institute	Metabolomics Platform	Broad Institute and Beth Israel Metabolomics Platform	Clary Clish	Cambridge	Massachusetts	2142	United States of America
Comhair, Suzy	Cleveland Clinic	Immunity and Immunology	PVDOMICS, SARP	Serpil Erzurum	Cleveland	Ohio	44195	United States of America
Comhair, Suzy	Cleveland Clinic	Immunity and Immunology	PVDOMICS, SARP	Deborah A Meyers	Cleveland	Ohio	44195	United States of America
Conomos, Matthew	University of Washington	Biostatistics			Seattle	Washington	98195	United States of America
Cornell, Elaine	University of Vermont		MESA	Jerome Rotter	Burlington	Vermont	5405	United States of America
Crandall, Carolyn	University of California, Los Angeles		WHI	Charles Kooperberg	Los Angeles	California	90095	United States of America
Crapo, James	National Jewish Health		COPD	Ed Silverman	Denver	Colorado	80206	United States of America
Cupples, L. Adrienne	Boston University	Biostatistics	FHS	Joanne Murabito	Boston	Massachusetts	2115	United States of America
Curran, Joanne	University of Texas Rio Grande Valley School of Medicine		SAFS	John Blangero	Brownsville	Texas	78520	United States of America
Curtis, Jeffrey	University of Michigan	Internal Medicine	COPD	Ed Silverman	Ann Arbor	Michigan	48109	United States of America
Custer, Brian	Vitalant Research Institute		REDS-III_Brazil	Brian Custer	San Francisco	California	94118	United States of America
Damcott, Coleen	University of Maryland		Amish	Braxton D. Mitchell	Baltimore	Maryland	21201	United States of America
Darbar, Dawood	University of Illinois at Chicago		AFGen	Patrick Ellinor	Chicago	Illinois	60607	United States of America
David, Sean	University of Chicago		WHI	Charles Kooperberg	Chicago	Illinois	60637	United States of America
Davis, Colleen	University of Washington		Northwest Genomics Center	Evan Eichler	Seattle	Washington	98195	United States of America
Daya, Michelle	University of Colorado at Denver		BAGS, GeneSTAR	Lisa Yanek	Denver	Colorado	80204	United States of America
Daya, Michelle	University of Colorado at Denver		BAGS, GeneSTAR	Kathleen Barnes	Denver	Colorado	80204	United States of America
de Andrade, Mariza	Mayo Clinic	Health Quantitative Sciences Research	HyperGEN_GENOA, VTE	Donna K Arnett	Rochester	Minnesota	55905	United States of America
de Andrade, Mariza	Mayo Clinic	Health Quantitative Sciences Research	HyperGEN_GENOA, VTE	Eric Boerwinkle	Rochester	Minnesota	55905	United States of America

de las Fuentes, Lisa	Washington University in St Louis	Department of Medicine, Cardiovascular Division	THRV	Yii-Der Ida Chen	St. Louis	Missouri	63110	United States of America
de Vries, Paul	University of Texas Health at Houston	Human Genetics Center, Department of Epidemiology, Human Genetics, and Environmental Sciences	VTE	Eric Boerwinkle	Houston	Texas	77030	United States of America
DeBaun, Michael	Vanderbilt University		HLKSCD, SIT_SCD	Michael DeBaun	Nashville	Tennessee	37235	United States of America
DeBaun, Michael	Vanderbilt University		HLKSCD, SIT_SCD	James Casella	Nashville	Tennessee	37235	United States of America
Deka, Ranjan	University of Cincinnati		Samoan	Ryan L Minster	Cincinnati	Ohio	45220	United States of America
DeMeo, Dawn	Brigham & Women's Hospital		COPD, CRA_CAMP, LTRC	Scott Weiss	Boston	Massachusetts	2115	United States of America
DeMeo, Dawn	Brigham & Women's Hospital		COPD, CRA_CAMP, LTRC	Ed Silverman	Boston	Massachusetts	2115	United States of America
DeMeo, Dawn	Brigham & Women's Hospital		COPD, CRA_CAMP, LTRC	Ed Silverman	Boston	Massachusetts	2115	United States of America
Devine, Scott	University of Maryland		Amish	Braxton D. Mitchell	Baltimore	Maryland	21201	United States of America
Dinh, Huyen	Baylor College of Medicine Human Genome Sequencing Center		Baylor	Richard Gibbs	Houston	Texas	77030	United States of America
Doddapaneni, Harsha	Baylor College of Medicine Human Genome Sequencing Center		Baylor	Richard Gibbs	Houston	Texas	77030	
Duan, Qing	University of North Carolina		JHS, WHI	Charles Kooperberg	Chapel Hill	North Carolina	27599	United States of America
Duan, Qing	University of North Carolina		JHS, WHI	Laura Raffield	Chapel Hill	North Carolina	27599	United States of America
Dugan-Perez, Shannon	Baylor College of Medicine Human Genome Sequencing Center	BCM	Baylor	Richard Gibbs	Houston	Texas	77030	United States of America
Duggirala, Ravi	University of Texas Rio Grande Valley School of Medicine		SAFS	John Blangero	Edinburg	Texas	78539	United States of America
Durda, Jon Peter	University of Vermont	Pathology and Laboratory Medicine	JHS, MESA, VTE	Laura Raffield	Burlington	Vermont	5405	United States of America
Durda, Jon Peter	University of Vermont	Pathology and Laboratory Medicine	JHS, MESA, VTE	Eric Boerwinkle	Burlington	Vermont	5405	United States of America
Durda, Jon Peter	University of Vermont	Pathology and Laboratory Medicine	JHS, MESA, VTE	Jerome Rotter	Burlington	Vermont	5405	United States of America
Dutcher, Susan K.	Washington University in St Louis	Genetics	MGI	Susan K. Dutcher	St Louis	Missouri	63110	United States of America
Eaton, Charles	Brown University		WHI	Charles Kooperberg	Providence	Rhode Island	2912	United States of America
Ekunwe, Lynette	University of Mississippi		JHS	Laura Raffield	Jackson	Mississippi	38677	United States of America
El Boueiz, Adel	Harvard University	Channing Division of Network Medicine	COPD, LTRC	Ed Silverman	Cambridge	Massachusetts	2138	United States of America
El Boueiz, Adel	Harvard University	Channing Division of Network Medicine	COPD, LTRC	Ed Silverman	Cambridge	Massachusetts	2138	United States of America

Ellinor, Patrick	Massachusetts General Hospital		AFGen, FHS	Patrick Ellinor	Boston	Massachusetts	2114	United States of America
Ellinor, Patrick	Massachusetts General Hospital		AFGen, FHS	Joanne Murabito	Boston	Massachusetts	2114	United States of America
Emery, Leslie	University of Washington				Seattle	Washington	98195	United States of America
Erzurum, Serpil	Cleveland Clinic	Lerner Research Institute	PVDOMICS, SARP	Serpil Erzurum	Cleveland	Ohio	44195	United States of America
Erzurum, Serpil	Cleveland Clinic	Lerner Research Institute	PVDOMICS, SARP	Deborah A Meyers	Cleveland	Ohio	44195	United States of America
Farber, Charles	University of Virginia		MESA	Jerome Rotter	Charlottesville	Virginia	22903	United States of America
Farek, Jesse	Baylor College of Medicine Human Genome Sequencing Center		Baylor	Richard Gibbs	Houston	Texas	77030	United States of America
Fingerlin, Tasha	National Jewish Health	Center for Genes, Environment and Health	IPF	David Schwartz	Denver	Colorado	80206	United States of America
Flickinger, Matthew	University of Michigan		IRC	Gonçalo Abecasis	Ann Arbor	Michigan	48109	United States of America
Fornage, Myriam	University of Texas Health at Houston		CARDIA, VTE	Myriam Fornage	Houston	Texas	77225	United States of America
Fornage, Myriam	University of Texas Health at Houston		CARDIA, VTE	Eric Boerwinkle	Houston	Texas	77225	United States of America
Franceschini, Nora	University of North Carolina	Epidemiology	VTE, WHI	Charles Kooperberg	Chapel Hill	North Carolina	27599	United States of America
Franceschini, Nora	University of North Carolina	Epidemiology	VTE, WHI	Eric Boerwinkle	Chapel Hill	North Carolina	27599	United States of America
Frazar, Chris	University of Washington		Northwest Genomics Center	Evan Eichler	Seattle	Washington	98195	United States of America
Fu, Mao	University of Maryland		Amish	Braxton D. Mitchell	Baltimore	Maryland	21201	United States of America
Fullerton, Stephanie M.	University of Washington				Seattle	Washington	98195	United States of America
Fulton, Lucinda	Washington University in St Louis		MGI	Susan K. Dutcher	St Louis	Missouri	63130	United States of America
Gabriel, Stacey	Broad Institute		Broad Institute Genomics Platform	Stacey Gabriel	Cambridge	Massachusetts	2142	United States of America
Gao, Shanshan	University of Colorado at Denver		TOPCHeF	Matthew Taylor	Denver	Colorado	80204	United States of America
Gao, Yan	University of Mississippi		JHS	Laura Raffield	Jackson	Mississippi	38677	United States of America
Gass, Margery	Fred Hutchinson Cancer Research Center		WHI	Charles Kooperberg	Seattle	Washington	98109	United States of America
Geiger, Heather	New York Genome Center		New York Genome Center Genomics	Soren Germer	New York City	New York	10013	United States of America
Gelb, Bruce	Icahn School of Medicine at Mount Sinai		PCGC_CHD	Bruce Gelb	New York	New York	10029	United States of America
Geraci, Mark	University of Pittsburgh		PVDOMICS	Serpil Erzurum	Pittsburgh	Pennsylvania		United States of America
Germer, Soren	New York Genome Center		New York Genome Center Genomics	Soren Germer	New York	New York	10013	United States of America
Gerszten, Robert	Beth Israel Deaconess Medical Center		Broad Institute Genomics Platform, Broad Institute and Beth Israel Metabolomics Platform, Broad Institute and Beth	Jerome Rotter	Boston	Massachusetts	2215	United States of America

			Israel Proteomics Platform, JHS, MESA					
Gerszten, Robert	Beth Israel Deaconess Medical Center		Broad Institute Genomics Platform, Broad Institute and Beth Israel Metabolomics Platform, Broad Institute and Beth Israel Proteomics Platform, JHS, MESA	Robert Gerszten	Boston	Massachusetts	2215	United States of America
Gerszten, Robert	Beth Israel Deaconess Medical Center		Broad Institute Genomics Platform, Broad Institute and Beth Israel Metabolomics Platform, Broad Institute and Beth Israel Proteomics Platform, JHS, MESA	Clary Clish	Boston	Massachusetts	2215	United States of America
Gerszten, Robert	Beth Israel Deaconess Medical Center		Broad Institute Genomics Platform, Broad Institute and Beth Israel Metabolomics Platform, Broad Institute and Beth Israel Proteomics Platform, JHS, MESA	Stacey Gabriel	Boston	Massachusetts	2215	United States of America
Gerszten, Robert	Beth Israel Deaconess Medical Center		Broad Institute Genomics Platform, Broad Institute and Beth Israel Metabolomics Platform, Broad Institute and Beth Israel Proteomics Platform, JHS, MESA	Laura Raffield	Boston	Massachusetts	2215	United States of America
Ghosh, Auyon	Brigham & Women's Hospital		COPD, LTRC	Ed Silverman	Boston	Massachusetts	2115	United States of America
Ghosh, Auyon	Brigham & Women's Hospital		COPD, LTRC	Ed Silverman	Boston	Massachusetts	2115	United States of America
Gibbs, Richard	Baylor College of Medicine Human Genome Sequencing Center		Baylor	Richard Gibbs	Houston	Texas	77030	United States of America
Gignoux, Chris	Stanford University		BAGS	Kathleen Barnes	Stanford	California	94305	United States of America
Gladwin, Mark	University of Pittsburgh		walk_PHaSST	Victor Gordeuk	Pittsburgh	Pennsylvania	15260	United States of America
Glahn, David	Boston Children's Hospital, Harvard Medical School	Department of Psychiatry	SAFS	John Blangero	Boston	Massachusetts	2115	United States of America
Gogarten, Stephanie	University of Washington				Seattle	Washington	98195	United States of America
Gong, Da-Wei	University of Maryland		Amish	Braxton D. Mitchell	Baltimore	Maryland	21201	United States of America
Goring, Harald	University of Texas Rio Grande Valley School of Medicine		SAFS	John Blangero	San Antonio	Texas	78229	United States of America
Graw, Sharon	University of Colorado Anschutz Medical Campus		TOPCHeF	Matthew Taylor	Aurora	Colorado	80045	United States of America
Gray, Kathryn J.	University of Washington	Obstetrics and Gynecology	BCC-PREG	Kathryn J. Gray	Seattle	Washington	98195	United States of America
Grine, Daniel	University of Colorado at Denver		TOPCHeF	Matthew Taylor	Denver	Colorado	80204	United States of America

Gross, Colin	University of Michigan		IRC	Gonçalo Abecasis	Ann Arbor	Michigan	48109	United States of America
Gu, C. Charles	Washington University in St Louis	Institute for Informatics, Data Science and Biostatistics	HyperGEN_GENOA	Donna K Arnett	St Louis	Missouri	63130	United States of America
Guan, Yue	University of Maryland		Amish	Braxton D. Mitchell	Baltimore	Maryland	21201	United States of America
Guo, Xiuqing	Lundquist Institute		AA_CAC, MESA, THRV	Kent D. Taylor	Torrance	California	90502	United States of America
Guo, Xiuqing	Lundquist Institute		AA_CAC, MESA, THRV	Jerome Rotter	Torrance	California	90502	United States of America
Guo, Xiuqing	Lundquist Institute		AA_CAC, MESA, THRV	Yii-Der Ida Chen	Torrance	California	90502	United States of America
Gupta, Namrata	Broad Institute		Broad Institute Genomics Platform	Stacey Gabriel	Cambridge	Massachusetts	2142	United States of America
Haessler, Jeff	Fred Hutchinson Cancer Research Center		WHI	Charles Kooperberg	Seattle	Washington	98109	United States of America
Hall, Michael	University of Mississippi	Cardiology	JHS	Laura Raffield	Jackson	Mississippi	39216	United States of America
Han, Yi	Baylor College of Medicine Human Genome Sequencing Center		Baylor	Richard Gibbs	Houston	Texas	77030	United States of America
Hanly, Patrick	University of Calgary	Medicine	GEM-OSA	Allan Pack	Calgary			Canada
Harris, Daniel	University of Maryland	Genetics	Amish	Braxton D. Mitchell	Philadelphia	Pennsylvania	19104	United States of America
Hawley, Nicola L.	Yale University	Department of Chronic Disease Epidemiology	Samoan	Ryan L Minster	New Haven	Connecticut	6520	United States of America
He, Jiang	Tulane University		GenSalt	Jiang He	New Orleans	Louisiana	70118	United States of America
Heavner, Ben	University of Washington	Biostatistics			Seattle	Washington	98195	United States of America
Heckbert, Susan	University of Washington	Epidemiology	AFGen, CHS, MESA, VTE	Patrick Ellinor	Seattle	Washington	98195	United States of America
Heckbert, Susan	University of Washington	Epidemiology	AFGen, CHS, MESA, VTE	Jerome Rotter	Seattle	Washington	98195	United States of America
Heckbert, Susan	University of Washington	Epidemiology	AFGen, CHS, MESA, VTE	Bruce Psaty	Seattle	Washington	98195	United States of America
Heckbert, Susan	University of Washington	Epidemiology	AFGen, CHS, MESA, VTE	Eric Boerwinkle	Seattle	Washington	98195	United States of America
Hernandez, Ryan	University of California, San Francisco		PGX_Asthma	Esteban Burchard	San Francisco	California	94143	United States of America
Herrington, David	Wake Forest Baptist Health		MESA	Jerome Rotter	Winston-Salem	North Carolina	27157	United States of America
Hersh, Craig	Brigham & Women's Hospital	Channing Division of Network Medicine	COPD, LTRC	Ed Silverman	Boston	Massachusetts	2115	United States of America
Hersh, Craig	Brigham & Women's Hospital	Channing Division of Network Medicine	COPD, LTRC	Ed Silverman	Boston	Massachusetts	2115	United States of America
Hidalgo, Bertha	University of Alabama		GOLDN	Donna K Arnett	Birmingham	Alabama	35487	United States of America
Hixson, James	University of Texas Health at Houston		GenSalt, THRV	Yii-Der Ida Chen	Houston	Texas	77225	United States of America
Hixson, James	University of Texas Health at Houston		GenSalt, THRV	Jiang He	Houston	Texas	77225	United States of America

Hobbs, Brian	Brigham & Women's Hospital		COPD, LTRC	Ed Silverman	Boston	Massachusetts	2115	United States of America
Hobbs, Brian	Brigham & Women's Hospital		COPD, LTRC	Ed Silverman	Boston	Massachusetts	2115	United States of America
Hokanson, John	University of Colorado at Denver		COPD	Ed Silverman	Denver	Colorado	80204	United States of America
Hong, Elliott	University of Maryland		Amish	Braxton D. Mitchell	Baltimore	Maryland	21201	United States of America
Hoth, Karin	University of Iowa		COPD	Ed Silverman	Iowa City	Iowa	52242	United States of America
Hsiung, Chao (Agnes)	National Health Research Institute Taiwan	Institute of Population Health Sciences, NHRI	THRV	Yii-Der Ida Chen	Miaoli County		350	Taiwan (Province of China)
Hu, Jianhong	Baylor College of Medicine Human Genome Sequencing Center		Baylor	Richard Gibbs	Houston	Texas	77030	United States of America
Hung, Yi-Jen	Tri-Service General Hospital National Defense Medical Center		THRV	Yii-Der Ida Chen				Taiwan (Province of China)
Huston, Haley	Blood Works Northwest		MLOF	Barbara Konkle	Seattle	Washington	98104	United States of America
Hwu, Chii Min	Taichung Veterans General Hospital Taiwan		THRV	Yii-Der Ida Chen	Taichung City		407	Taiwan (Province of China)
Irvin, Marguerite Ryan	University of Alabama		GOLDN, HyperGEN_GENOA	Donna K Arnett	Birmingham	Alabama	35487	United States of America
Irvin, Marguerite Ryan	University of Alabama		GOLDN, HyperGEN_GENOA	Donna K Arnett	Birmingham	Alabama	35487	United States of America
Jackson, Rebecca	Oklahoma State University Medical Center	Internal Medicine, DIvision of Endocrinology, Diabetes and Metabolism	WHI	Charles Kooperberg	Columbus	Ohio	43210	United States of America
Jain, Deepti	University of Washington				Seattle	Washington	98195	United States of America
Jaquish, Cashell	National Heart, Lung, and Blood Institute, National Institutes of Health	NHLBI	Amish, NHLBI Program	Braxton D. Mitchell	Bethesda	Maryland	20892	United States of America
Johnsen, Jill	University of Washington	Medicine	HIPS, MLOF	Deborah Brown	Seattle	Washington	98109	United States of America
Johnsen, Jill	University of Washington	Medicine	HIPS, MLOF	Barbara Konkle	Seattle	Washington	98109	United States of America
Johnson, Andrew	National Heart, Lung, and Blood Institute, National Institutes of Health		FHS	Joanne Murabito	Bethesda	Maryland	20892	United States of America
Johnson, Craig	University of Washington		MESA	Jerome Rotter	Seattle	Washington	98195	United States of America
Johnston, Rich	Emory University		BAGS	Kathleen Barnes	Atlanta	Georgia	30322	United States of America
Jones, Kimberly	Johns Hopkins University		SIT_SCD	James Casella	Baltimore	Maryland	21218	United States of America
Kaplan, Robert	Albert Einstein College of Medicine		HCHS_SOL	Robert Kaplan	New York	New York	10461	United States of America
Kardia, Sharon	University of Michigan		AA_CAC, HyperGEN_GENOA	Kent D. Taylor	Ann Arbor	Michigan	48109	United States of America
Kardia, Sharon	University of Michigan		AA_CAC, HyperGEN_GENOA	Donna K Arnett	Ann Arbor	Michigan	48109	United States of America
Kelly, Shannon	University of California, San Francisco		REDS-III_Brazil	Brian Custer	San Francisco	California	94118	United States of America

Kenny, Eimear	Icahn School of Medicine at Mount Sinai		BioMe	Ruth J.F. Loos	New York	New York	10029	United States of America
Kessler, Michael	University of Maryland		Amish	Braxton D. Mitchell	Baltimore	Maryland	21201	United States of America
Khan, Alyna	University of Washington				Seattle	Washington	98195	United States of America
Khan, Ziad	Baylor College of Medicine Human Genome Sequencing Center		Baylor	Richard Gibbs	Houston	Texas	77030	United States of America
Kim, Wonji	Harvard University		COPD, ECLIPSE	Ed Silverman	Cambridge	Massachusetts	2138	United States of America
Kim, Wonji	Harvard University		COPD, ECLIPSE	Ed Silverman	Cambridge	Massachusetts	2138	United States of America
Kimoff, John	McGill University		GEM-OSA	Allan Pack	Montr 챕 al		QC H3A 0G4	Canada
Kinney, Greg	University of Colorado at Denver	Epidemiology	COPD	Ed Silverman	Aurora	Colorado	80045	United States of America
Konkle, Barbara	Blood Works Northwest	Medicine	MLOF	Barbara Konkle	Seattle	Washington	98104	United States of America
Kooperberg, Charles	Fred Hutchinson Cancer Research Center		Analysis Program, WHI	Charles Kooperberg	Seattle	Washington	98109	United States of America
Kramer, Holly	Loyola University	Public Health Sciences	MESA	Jerome Rotter	Maywood	Illinois	60153	United States of America
Lange, Christoph	Harvard School of Public Health	Biostats	COPD, CRA_CAMP	Scott Weiss	Boston	Massachusetts	2115	United States of America
Lange, Christoph	Harvard School of Public Health	Biostats	COPD, CRA_CAMP	Ed Silverman	Boston	Massachusetts	2115	United States of America
Lange, Ethan	University of Colorado at Denver		JHS	Laura Raffield	Denver	Colorado	80204	United States of America
Laurie, Cathy	University of Washington		HCHS_SOL	Robert Kaplan	Seattle	Washington	98195	United States of America
Laurie, Cathy	University of Washington		HCHS_SOL		Seattle	Washington	98195	United States of America
Laurie, Cecelia	University of Washington				Seattle	Washington	98195	United States of America
LeBoff, Meryl	Brigham & Women's Hospital		WHI	Charles Kooperberg	Boston	Massachusetts	2115	United States of America
Lee, Jiwon	Brigham & Women's Hospital		CFS	Susan Redline	Boston	Massachusetts	2115	United States of America
Lee, Sandra	Baylor College of Medicine Human Genome Sequencing Center		Baylor	Richard Gibbs	Houston	Texas	77030	United States of America
Lee, Wen-Jane	Taichung Veterans General Hospital Taiwan		THRV	Yii-Der Ida Chen	Taichung City		407	Taiwan (Province of China)
LeFaive, Jonathon	University of Michigan		IRC	Gonçalo Abecasis	Ann Arbor	Michigan	48109	United States of America
Levine, David	University of Washington				Seattle	Washington	98195	United States of America
Levy, Dan	National Heart, Lung, and Blood Institute, National Institutes of Health		FHS	Joanne Murabito	Bethesda	Maryland	20892	United States of America
Lewis, Joshua	University of Maryland		Amish	Braxton D. Mitchell	Baltimore	Maryland	21201	United States of America
Li, Xiaohui	Lundquist Institute		MESA	Jerome Rotter	Torrance	California	90502	United States of America
Li, Yun	University of North Carolina		JHS, MESA, WHI	Jerome Rotter	Chapel Hill	North Carolina	27599	United States of America
Li, Yun	University of North Carolina		JHS, MESA, WHI	Charles Kooperberg	Chapel Hill	North Carolina	27599	United States of America

Li, Yun	University of North Carolina		JHS, MESA, WHI	Laura Raffield	Chapel Hill	North Carolina	27599	United States of America
Lin, Henry	Lundquist Institute		MESA	Jerome Rotter	Torrance	California	90502	United States of America
Lin, Honghuang	Boston University	University of Massachusetts Chan Medical School	FHS	Joanne Murabito	Worcester	Massachusetts	1655	United States of America
Lin, Xihong	Harvard School of Public Health	Biostatistics	CFS	Susan Redline	Boston	Massachusetts	2115	United States of America
Liu, Simin	Brown University	Epidemiology and Medicine	JHS, WHI	Charles Kooperberg	Providence	Rhode Island	2912	United States of America
Liu, Simin	Brown University	Epidemiology and Medicine	JHS, WHI	Laura Raffield	Providence	Rhode Island	2912	United States of America
Liu, Yongmei	Duke University	Cardiology	MESA	Jerome Rotter	Durham	North Carolina	27708	United States of America
Liu, Yu	Stanford University	Cardiovascular Institute	SCVI	Joseph Wu	Stanford	California	94305	United States of America
Loos, Ruth J.F.	Icahn School of Medicine at Mount Sinai	The Charles Bronfman Institute for Personalized Medicine	BioMe	Ruth J.F. Loos	New York	New York	10029	United States of America
Lubitz, Steven	Massachusetts General Hospital		AFGen, FHS	Patrick Ellinor	Boston	Massachusetts	2114	United States of America
Lubitz, Steven	Massachusetts General Hospital		AFGen, FHS	Joanne Murabito	Boston	Massachusetts	2114	United States of America
Lunetta, Kathryn	Boston University		AFGen	Patrick Ellinor	Boston	Massachusetts	2215	United States of America
Magalang, Ulysses	The Ohio State University	Division of Pulmonary, Critical Care and Sleep Medicine	GEM-OSA	Allan Pack	Columbus	Ohio	43210	United States of America
Mahaney, Michael	University of Texas Rio Grande Valley School of Medicine		SAFS	John Blangero	Brownsville	Texas	78520	United States of America
Make, Barry	Johns Hopkins University		COPD	Ed Silverman	Baltimore	Maryland	21218	United States of America
Manichaikul, Ani	University of Virginia		Analysis Program, MESA	Jerome Rotter	Charlottesville	Virginia	22903	United States of America
Manning, Alisa	Broad Institute, Harvard University, Massachusetts General Hospital		Broad Institute Genomics Platform, FHS	Joanne Murabito				
Manning, Alisa	Broad Institute, Harvard University, Massachusetts General Hospital		Broad Institute Genomics Platform, FHS	Stacey Gabriel				
Manson, JoAnn	Brigham & Women's Hospital		WHI	Charles Kooperberg	Boston	Massachusetts	2115	United States of America
Martin, Lisa	George Washington University	cardiology	WHI	Charles Kooperberg	Washington	District of Columbia	20037	United States of America
Marton, Melissa	New York Genome Center		New York Genome Center Genomics	Soren Germer	New York City	New York	10013	United States of America
Mathai, Susan	University of Colorado at Denver		IPF	David Schwartz	Denver	Colorado	80204	United States of America
Mathias, Rasika	National Institutes of Health		Analysis Program, BAGS, GeneSTAR	Lisa Yanek	Rockville	Maryland	20852	United States of America
Mathias, Rasika	National Institutes of Health		Analysis Program, BAGS, GeneSTAR	Kathleen Barnes	Rockville	Maryland	20852	United States of America
May, Susanne	University of Washington	Biostatistics			Seattle	Washington	98195	United States of America

McArdle, Patrick	University of Maryland		Amish	Braxton D. Mitchell	Baltimore	Maryland	21201	United States of America
McDonald, Merry-Lynn	University of Alabama	University of Alabama at Birmingham	COPD	Ed Silverman	Birmingham	Alabama	35487	United States of America
McFarland, Sean	Harvard University		Boston-Brazil_SCD	Vijay G. Sankaran	Cambridge	Massachusetts	2138	United States of America
McGarvey, Stephen	Brown University	Epidemiology	Samoan	Ryan L Minster	Providence	Rhode Island	2912	United States of America
McGoldrick, Daniel	University of Washington	Genome Sciences	MESA, Northwest Genomics Center	Jerome Rotter	Seattle	Washington	98195	United States of America
McGoldrick, Daniel	University of Washington	Genome Sciences	MESA, Northwest Genomics Center	Evan Eichler	Seattle	Washington	98195	United States of America
McHugh, Caitlin	University of Washington	Biostatistics			Seattle	Washington	98195	United States of America
McNeil, Becky	RTI International		nuMoM2b-HHS	Nathan Blue				United States of America
Mei, Hao	University of Mississippi		JHS	Laura Raffield	Jackson	Mississippi	38677	United States of America
Meigs, James	Massachusetts General Hospital	Medicine	FHS	Joanne Murabito	Boston	Massachusetts	2114	United States of America
Menon, Vipin	Baylor College of Medicine Human Genome Sequencing Center		Baylor	Richard Gibbs	Houston	Texas	77030	United States of America
Mestroni, Luisa	University of Colorado Anschutz Medical Campus		TOPCHeF	Matthew Taylor	Aurora	Colorado	80045	United States of America
Metcalf, Ginger	Baylor College of Medicine Human Genome Sequencing Center		Baylor	Richard Gibbs	Houston	Texas	77030	United States of America
Meyers, Deborah A	Mayo Clinic		COPDMet, SARP, SPIROMICS	Russell Bowler				United States of America
Meyers, Deborah A	Mayo Clinic		COPDMet, SARP, SPIROMICS	Deborah A Meyers				United States of America
Meyers, Deborah A	Mayo Clinic		COPDMet, SARP, SPIROMICS	Deborah A Meyers				United States of America
Mignot, Emmanuel	Stanford University	Center For Sleep Sciences and Medicine	GEM-OSA	Allan Pack	Palo Alto	California	94304	United States of America
Min, Yuan-I	University of Mississippi		JHS	Laura Raffield	Jackson	Mississippi	39213	United States of America
Minster, Ryan L	University of Pittsburgh		Samoan	Ryan L Minster	Pittsburgh	Pennsylvania	15260	United States of America
Mitchell, Braxton D.	University of Maryland		Amish	Braxton D. Mitchell	Baltimore	Maryland	21201	United States of America
Moll, Matt	Brigham & Women's Hospital	Medicine	COPD	Ed Silverman	Boston	Massachusetts	2115	United States of America
Momin, Zeineen	Baylor College of Medicine Human Genome Sequencing Center		Baylor	Richard Gibbs	Houston	Texas	77030	United States of America
Montasser, May	National Heart, Lung, and Blood Institute		Amish, Analysis Program	Braxton D. Mitchell	Bethesda	Maryland	20817	United States of America
Montgomery, Courtney	Oklahoma Medical Research Foundation	Genes and Human Disease	Sarcoidosis	Courtney Montgomery	Oklahoma City	Oklahoma	73104	United States of America
Muzny, Donna	Baylor College of Medicine Human Genome Sequencing Center		Baylor	Richard Gibbs	Houston	Texas	77030	United States of America
Mychaleckyj, Josyf C	University of Virginia		MESA	Jerome Rotter	Charlottesville	Virginia	22903	United States of America
Nadkarni, Girish	Icahn School of Medicine at Mount Sinai		BioMe	Ruth J.F. Loos	New York	New York	10029	United States of America

Naik, Rakhi	Johns Hopkins University		JHS	Laura Raffield	Baltimore	Maryland	21218	United States of America
Naseri, Take	Ministry of Health, Government of Samoa		Samoan	Ryan L Minster	Apia			Samoa
Natarajan, Pradeep	Broad Institute		Analysis Program, Broad Institute Genomics Platform	Stacey Gabriel	Cambridge	Massachusetts	2142	United States of America
Nekhai, Sergei	Howard University		PUSH_SCD	Sergei Nekhai	Washington	District of Columbia	20059	United States of America
Nelson, Sarah C.	University of Washington	Biostatistics			Seattle	Washington	98195	United States of America
Neltner, Bonnie	University of Colorado at Denver		TOPCHeF	Matthew Taylor	Denver	Colorado	80204	United States of America
Nessner, Caitlin	Baylor College of Medicine Human Genome Sequencing Center		Baylor	Richard Gibbs	Houston	Texas	77030	United States of America
Nickerson, Deborah	University of Washington	Department of Genome Sciences	Northwest Genomics Center	Evan Eichler	Seattle	Washington	98195	United States of America
Nkechinyere, Osuji	Baylor College of Medicine Human Genome Sequencing Center		Baylor	Richard Gibbs	Houston	Texas	77030	United States of America
North, Kari	University of North Carolina		HCHS_SOL, VTE, WHI	Charles Kooperberg	Chapel Hill	North Carolina	27599	United States of America
North, Kari	University of North Carolina		HCHS_SOL, VTE, WHI	Eric Boerwinkle	Chapel Hill	North Carolina	27599	United States of America
North, Kari	University of North Carolina		HCHS_SOL, VTE, WHI	Robert Kaplan	Chapel Hill	North Carolina	27599	United States of America
O'Connell, Jeff	University of Maryland		Amish, Analysis Program	Braxton D. Mitchell	Balitmore	Maryland	21201	United States of America
O'Connor, Tim	University of Maryland		Amish, Analysis Program	Braxton D. Mitchell	Baltimore	Maryland	21201	United States of America
Ochs-Balcom, Heather	University at Buffalo		WHI	Charles Kooperberg	Buffalo	New York	14260	United States of America
Okwuonu, Geoffrey	Baylor College of Medicine Human Genome Sequencing Center		Baylor	Richard Gibbs	Houston	Texas	77030	United States of America
Pack, Allan	University of Pennsylvania	Division of Sleep Medicine/Department of Medicine	GEM-OSA	Allan Pack	Philadelphia	Pennsylvania	19104-3403	United States of America
Paik, David T.	Stanford University	Stanford Cardiovascular Institute	SCVI	Joseph Wu	Stanford	California	94305	United States of America
Palmer, Nicholette	Wake Forest Baptist Health	Biochemistry	AA_CAC	Kent D. Taylor	Winston-Salem	North Carolina	27157	United States of America
Pankow, James	University of Minnesota		MESA, VTE	Jerome Rotter	Minneapolis	Minnesota	55455	United States of America
Pankow, James	University of Minnesota		MESA, VTE	Eric Boerwinkle	Minneapolis	Minnesota	55455	United States of America
Parker, Cora	RTI International	Biostatistics and Epidemiology Division	nuMoM2b-HHS	Nathan Blue	Research Triangle Park	North Carolina	27709-2194	United States of America
Peloso, Gina	Boston University	Department of Biostatistics	Analysis Program, FHS	Joanne Murabito	Boston	Massachusetts	2118	United States of America
Peralta, Juan Manuel	University of Texas Rio Grande Valley School of Medicine		SAFS	John Blangero	Edinburg	Texas	78539	United States of America
Perez, Marco	Stanford University		WHI	Charles Kooperberg	Stanford	California	94305	United States of America
Perry, James	University of Maryland		Amish, Analysis Program	Braxton D. Mitchell	Baltimore	Maryland	21201	United States of America

Peters, Ulrike	Fred Hutchinson Cancer Research Center	Fred Hutch and UW	WHI	Charles Kooperberg	Seattle	Washington	98109	United States of America
Peyser, Patricia	University of Michigan		AA_CAC, HyperGEN_GENOA	Donna K Arnett	Ann Arbor	Michigan	48109	United States of America
Peyser, Patricia	University of Michigan		AA_CAC, HyperGEN_GENOA	Kent D. Taylor	Ann Arbor	Michigan	48109	United States of America
Phillips, Lawrence S	Emory University		WHI	Charles Kooperberg	Atlanta	Georgia	30322	United States of America
Pleiness, Jacob	University of Michigan		IRC	Gonçalo Abecasis	Ann Arbor	Michigan	48109	United States of America
Pollin, Toni	University of Maryland		Amish	Braxton D. Mitchell	Baltimore	Maryland	21201	United States of America
Post, Wendy	Johns Hopkins University	Cardiology/Medicine	MESA	Jerome Rotter	Baltimore	Maryland	21218	United States of America
Powers Becker, Julia	University of Colorado at Denver	Medicine	IPF	David Schwartz	Denver	Colorado	80204	United States of America
Preethi Boorgula, Meher	University of Colorado at Denver		BAGS	Kathleen Barnes	Denver	Colorado	80204	United States of America
Preuss, Michael	Icahn School of Medicine at Mount Sinai		BioMe	Ruth J.F. Loos	New York	New York	10029	United States of America
Psaty, Bruce	University of Washington		CHS	Bruce Psaty	Seattle	Washington	98195	United States of America
Qiao, Dandi	Brigham & Women's Hospital		COPD, ECLIPSE	Ed Silverman	Boston	Massachusetts	2115	United States of America
Qiao, Dandi	Brigham & Women's Hospital		COPD, ECLIPSE	Ed Silverman	Boston	Massachusetts	2115	United States of America
Qin, Zhaohui	Emory University		BAGS	Kathleen Barnes	Atlanta	Georgia	30322	United States of America
Rafaels, Nicholas	University of Colorado at Denver	CCPM	BAGS	Kathleen Barnes	Denver	Colorado	80045	United States of America
Raffield, Laura	University of North Carolina	Genetics	JHS, MESA	Laura Raffield	Chapel Hill	North Carolina	27599	United States of America
Raffield, Laura	University of North Carolina	Genetics	JHS, MESA	Jerome Rotter	Chapel Hill	North Carolina	27599	United States of America
Rajendran, Mahitha	Baylor College of Medicine Human Genome Sequencing Center		Baylor	Richard Gibbs	Houston	Texas	77030	United States of America
Rao, D.C.	Washington University in St Louis		HyperGEN_GENOA, THRV	Yii-Der Ida Chen	St Louis	Missouri	63130	United States of America
Rao, D.C.	Washington University in St Louis		HyperGEN_GENOA, THRV	Donna K Arnett	St Louis	Missouri	63130	United States of America
Rasmussen-Torvik, Laura	Northwestern University		MESA	Jerome Rotter	Chicago	Illinois	60208	United States of America
Ratan, Aakrosh	University of Virginia		MESA	Jerome Rotter	Charlottesville	Virginia	22903	United States of America
Redline, Susan	Brigham & Women's Hospital	Medicine	CFS, MESA	Jerome Rotter	Boston	Massachusetts	2115	United States of America
Redline, Susan	Brigham & Women's Hospital	Medicine	CFS, MESA	Susan Redline	Boston	Massachusetts	2115	United States of America
Reed, Robert	University of Maryland		Amish	Braxton D. Mitchell	Baltimore	Maryland	21201	United States of America
Reeves, Catherine	New York Genome Center	New York Genome Center	New York Genome Center Genomics, New York Genome Center RNA-seq	Soren Germer	New York City	New York	10013	United States of America

Reeves, Catherine	New York Genome Center	New York Genome Center	New York Genome Center Genomics, New York Genome Center RNA-seq	Nicolas Robine	New York City	New York	10013	United States of America
Regan, Elizabeth	National Jewish Health		COPD	Ed Silverman	Denver	Colorado	80206	United States of America
Reiner, Alex	Fred Hutchinson Cancer Research Center, University of Washington		Analysis Program, JHS, WHI, MLOF	Charles Kooperberg	Seattle	Washington	98109	United States of America
Reiner, Alex	Fred Hutchinson Cancer Research Center, University of Washington		Analysis Program, JHS, WHI, MLOF	Laura Raffield	Seattle	Washington	98109	United States of America
Reiner, Alex	Fred Hutchinson Cancer Research Center, University of Washington		Analysis Program, JHS, WHI, MLOF	Barbara Konkle	Seattle	Washington	98109	United States of America
Reupena, Muagututi?쁝 Sefuiva	Lutia I Puava Ae Mapu I Fagalele		Samoan	Ryan L Minster	Apia			Samoa
Robillard, Rebecca	University of Ottawa	Sleep Research Unit, University of Ottawa Institute for Mental Health Research	GEM-OSA	Allan Pack	Ottawa		ON K1Z 7K4	Canada
Robine, Nicolas	New York Genome Center		New York Genome Center Genomics, New York Genome Center RNA-seq	Soren Germer	New York City	New York	10013	United States of America
Robine, Nicolas	New York Genome Center		New York Genome Center Genomics, New York Genome Center RNA-seq	Nicolas Robine	New York City	New York	10013	United States of America
Roden, Dan	Vanderbilt University	Medicine, Pharmacology, Biomedicla Informatics	AFGen	Patrick Ellinor	Nashville	Tennessee	37235	United States of America
Roselli, Carolina	Broad Institute		AFGen	Patrick Ellinor	Cambridge	Massachusetts	2142	United States of America
Rotter, Jerome	Lundquist Institute	Pediatrics	AA_CAC, MESA, THRV	Kent D. Taylor	Torrance	California	90502	United States of America
Rotter, Jerome	Lundquist Institute	Pediatrics	AA_CAC, MESA, THRV	Jerome Rotter	Torrance	California	90502	United States of America
Rotter, Jerome	Lundquist Institute	Pediatrics	AA_CAC, MESA, THRV	Yii-Der Ida Chen	Torrance	California	90502	United States of America
Ruczinski, Ingo	Johns Hopkins University		BAGS, COPD	Ed Silverman	Baltimore	Maryland	21218	United States of America
Ruczinski, Ingo	Johns Hopkins University		BAGS, COPD	Kathleen Barnes	Baltimore	Maryland	21218	United States of America
Runnels, Alexi	New York Genome Center		New York Genome Center Genomics	Soren Germer	New York City	New York	10013	United States of America
Russell, Pamela	University of Colorado at Denver		IPF	David Schwartz	Denver	Colorado	80204	United States of America
Ruuska, Sarah	Blood Works Northwest		MLOF	Barbara Konkle	Seattle	Washington	98104	United States of America
Ryan, Kathleen	University of Maryland		Amish	Braxton D. Mitchell	Baltimore	Maryland	21201	United States of America
Sabino, Ester Cerdeira	Universidade de Sao Paulo	Faculdade de Medicina	REDS-III_Brazil	Brian Custer	Sao Paulo		1310000	Brazil
Saleheen, Danish	Columbia University		PROMIS	Danish Saleheen	New York	New York	10027	United States of America
Salimi, Shabnam	University of Maryland	Pathology	Amish	Braxton D. Mitchell	Seattle	Washington	98195	United States of America

Salvi, Sejal	Baylor College of Medicine Human Genome Sequencing Center		Baylor	Richard Gibbs	Houston	Texas	77030	United States of America
Salzberg, Steven	Johns Hopkins University		BAGS	Kathleen Barnes	Baltimore	Maryland	21218	United States of America
Sandow, Kevin	Lundquist Institute	TGPS	THRV	Yii-Der Ida Chen	Torrance	California	90502	United States of America
Sankaran, Vijay G.	Harvard University	Division of Hematology/Oncology	Boston-Brazil_SCD	Vijay G. Sankaran	Boston	Massachusetts	2115	United States of America
Santibanez, Jireh	Baylor College of Medicine Human Genome Sequencing Center		Baylor	Richard Gibbs	Houston	Texas	77030	United States of America
Schwander, Karen	Washington University in St Louis		THRV	Yii-Der Ida Chen	St Louis	Missouri	63130	United States of America
Schwartz, David	University of Colorado at Denver		IPF	David Schwartz	Denver	Colorado	80204	United States of America
Sciurba, Frank	University of Pittsburgh		Analysis Program, COPD, LTRC	Ed Silverman	Pittsburgh	Pennsylvania	15260	United States of America
Sciurba, Frank	University of Pittsburgh		Analysis Program, COPD, LTRC	Ed Silverman	Pittsburgh	Pennsylvania	15260	United States of America
Seidman, Christine	Harvard Medical School	Genetics	PCGC_CHD, UNID_CM	Jonathan Seidman	Boston	Massachusetts	2115	United States of America
Seidman, Christine	Harvard Medical School	Genetics	PCGC_CHD, UNID_CM	Bruce Gelb	Boston	Massachusetts	2115	United States of America
Seidman, Jonathan	Harvard Medical School		PCGC_CHD, UNID_CM	Jonathan Seidman	Boston	Massachusetts	2115	United States of America
Seidman, Jonathan	Harvard Medical School		PCGC_CHD, UNID_CM	Bruce Gelb	Boston	Massachusetts	2115	United States of America
Sériès, Frédéric	Université Laval		GEM-OSA	Allan Pack	Quebec City		G1V 0A6	Canada
Sheehan, Vivien	Emory University	Pediatrics	PharmHU	Eric Boerwinkle	Atlanta	Georgia	30307	United States of America
Sherman, Stephanie L.	Emory University	Human Genetics	DS_CHD	Stephanie L. Sherman	Atlanta	Georgia	30322	United States of America
Shetty, Amol	University of Maryland		Amish	Braxton D. Mitchell	Baltimore	Maryland	21201	United States of America
Shetty, Aniket	University of Colorado at Denver		BAGS	Kathleen Barnes	Denver	Colorado	80204	United States of America
Sheu, Wayne Hui-Heng	Taichung Veterans General Hospital Taiwan		THRV	Yii-Der Ida Chen	Taichung City		407	Taiwan (Province of China)
Shoemaker, M. Benjamin	Vanderbilt University	Medicine/Cardiology	AFGen	Patrick Ellinor	Nashville	Tennessee	37235	United States of America
Silver, Brian	UMass Memorial Medical Center		WHI	Charles Kooperberg	Worcester	Massachusetts	1655	United States of America
Skomro, Robert	University of Saskatchewan		GEM-OSA	Allan Pack	Saskatoon		SK S7N 5C9	Canada
Smith, Albert Vernon	University of Michigan		IRC	Gonçalo Abecasis				
Smith, Jennifer	University of Michigan		HyperGEN_GENOA	Donna K Arnett	Ann Arbor	Michigan	48109	United States of America
Smith, Josh	University of Washington		Northwest Genomics Center	Evan Eichler	Seattle	Washington	98195	United States of America
Smith, Nicholas	University of Washington	Epidemiology	CHS, VTE	Bruce Psaty	Seattle	Washington	98195	United States of America
Smith, Nicholas	University of Washington	Epidemiology	CHS, VTE	Eric Boerwinkle	Seattle	Washington	98195	United States of America
Smith, Tanja	New York Genome Center		New York Genome Center Genomics	Soren Germer	New York	New York	10013	United States of America

Smoller, Sylvia	Albert Einstein College of Medicine		WHI	Charles Kooperberg	New York	New York	10461	United States of America
Snively, Beverly	Wake Forest Baptist Health	Biostatistical Sciences	WHI	Charles Kooperberg	Winston-Salem	North Carolina	27157	United States of America
Sofer, Tamar	Beth Israel Deaconess Medical Center		CFS, HCHS_SOL, JHS, MESA	Susan Redline	Boston	Massachusetts	2115	United States of America
Sofer, Tamar	Beth Israel Deaconess Medical Center		CFS, HCHS_SOL, JHS, MESA	Laura Raffield	Boston	Massachusetts	2115	United States of America
Sofer, Tamar	Beth Israel Deaconess Medical Center		CFS, HCHS_SOL, JHS, MESA	Robert Kaplan	Boston	Massachusetts	2115	United States of America
Sofer, Tamar	Beth Israel Deaconess Medical Center		CFS, HCHS_SOL, JHS, MESA	Jerome Rotter	Boston	Massachusetts	2115	United States of America
Sotoodehnia, Nona	University of Washington		CHS, MESA	Jerome Rotter	Seattle	Washington	98195	United States of America
Sotoodehnia, Nona	University of Washington		CHS, MESA	Bruce Psaty	Seattle	Washington	98195	United States of America
Stilp, Adrienne M.	University of Washington				Seattle	Washington	98195	United States of America
Storm, Garrett	University of Colorado at Denver	Genomic Cardiology	TOPCHeF	Matthew Taylor	Aurora	Colorado	80045	United States of America
Streeten, Elizabeth	University of Maryland		Amish	Braxton D. Mitchell	Baltimore	Maryland	21201	United States of America
Su, Jessica Lasky	Brigham & Women's Hospital	Channing Department of Medicine	CRA_CAMP	Scott Weiss	Boston	Massachusetts	2115	United States of America
Sung, Yun Ju	Washington University in St Louis		THRV	Yii-Der Ida Chen	St Louis	Missouri	63130	United States of America
Sylvia, Jody	Brigham & Women's Hospital		COPD	Ed Silverman	Boston	Massachusetts	2115	United States of America
Szpiro, Adam	University of Washington				Seattle	Washington	98195	United States of America
Taliun, Daniel	University of Michigan		IRC	Gonçalo Abecasis	Ann Arbor	Michigan	48109	United States of America
Tang, Hua	Stanford University	Genetics	Analysis Program, WHI	Charles Kooperberg	Stanford	California	94305	United States of America
Taub, Margaret	Johns Hopkins University		BAGS, GeneSTAR	Lisa Yanek	Baltimore	Maryland	21218	United States of America
Taub, Margaret	Johns Hopkins University		BAGS, GeneSTAR	Kathleen Barnes	Baltimore	Maryland	21218	United States of America
Taylor, Kent D.	Lundquist Institute	Institute for Translational Genomics and Populations Sciences	AA_CAC, MESA, THRV	Yii-Der Ida Chen	Torrance	California	90502	United States of America
Taylor, Kent D.	Lundquist Institute	Institute for Translational Genomics and Populations Sciences	AA_CAC, MESA, THRV	Kent D. Taylor	Torrance	California	90502	United States of America
Taylor, Kent D.	Lundquist Institute	Institute for Translational Genomics and Populations Sciences	AA_CAC, MESA, THRV	Jerome Rotter	Torrance	California	90502	United States of America
Taylor, Matthew	University of Colorado Anschutz Medical Campus		TOPCHeF	Matthew Taylor	Aurora	Colorado	80045	United States of America
Taylor, Simeon	University of Maryland		Amish	Braxton D. Mitchell	Baltimore	Maryland	21201	United States of America
Telen, Marilyn	Duke University		OMG_SCD	Allison Ashley-Koch	Durham	North Carolina	27708	United States of America

Thornton, Timothy A.	University of Washington				Seattle	Washington	98195	United States of America
Threlkeld, Machiko	University of Washington	University of Washington, Department of Genome Sciences	Northwest Genomics Center	Evan Eichler	Seattle	Washington	98195	United States of America
Tinker, Lesley	Fred Hutchinson Cancer Research Center	Cancer Prevention Division of Public Health Sciences	WHI	Charles Kooperberg	Seattle	Washington	98109	United States of America
Tirschwell, David	University of Washington		WHI	Charles Kooperberg	Seattle	Washington	98195	United States of America
Tishkoff, Sarah	University of Pennsylvania	Genetics	Africa6K	Sarah Tishkoff	Philadelphia	Pennsylvania	19104	United States of America
Tiwari, Hemant	University of Alabama	Biostatistics	GOLDN, HyperGEN_GENOA	Donna K Arnett	Birmingham	Alabama	35487	United States of America
Tiwari, Hemant	University of Alabama	Biostatistics	GOLDN, HyperGEN_GENOA	Donna K Arnett	Birmingham	Alabama	35487	United States of America
Tong, Catherine	University of Washington	Department of Biostatistics	MESA	Jerome Rotter	Seattle	Washington	98195	United States of America
Tracy, Russell	University of Vermont	Pathology & Laboratory Medicine	CHS, MESA	Jerome Rotter	Burlington	Vermont	5405	United States of America
Tracy, Russell	University of Vermont	Pathology & Laboratory Medicine	CHS, MESA	Bruce Psaty	Burlington	Vermont	5405	United States of America
Tsai, Michael	University of Minnesota		MESA	Jerome Rotter	Minneapolis	Minnesota	55455	United States of America
Vaidya, Dhananjay	Johns Hopkins University		GeneSTAR	Lisa Yanek	Baltimore	Maryland	21218	United States of America
Van Den Berg, David	University of Southern California	USC Methylation Characterization Center	Keck MGC	David Van Den Berg	University of Southern California	California	90033	United States of America
VandeHaar, Peter	University of Michigan		IRC	Gonçalo Abecasis	Ann Arbor	Michigan	48109	United States of America
Vrieze, Scott	University of Minnesota		IRC	Gonçalo Abecasis	Minneapolis	Minnesota	55455	United States of America
Walker, Tarik	University of Colorado at Denver		IPF	David Schwartz	Denver	Colorado	80204	United States of America
Wallace, Robert	University of Iowa		WHI	Charles Kooperberg	Iowa City	Iowa	52242	United States of America
Walts, Avram	University of Colorado at Denver		IPF	David Schwartz	Denver	Colorado	80204	United States of America
Wang, Fei Fei	University of Washington				Seattle	Washington	98195	United States of America
Wang, Heming	Brigham & Women's Hospital, Mass General Brigham		CFS	Susan Redline	Boston	Massachusetts	2115	United States of America
Wang, Jiongming	University of Michigan		IRC	Gonçalo Abecasis				United States of America
Watson, Karol	University of California, Los Angeles		MESA	Jerome Rotter	Los Angeles	California	90095	United States of America
Watt, Jennifer	Baylor College of Medicine Human Genome Sequencing Center		Baylor	Richard Gibbs	Houston	Texas	77030	United States of America
Weeks, Daniel E.	University of Pittsburgh	Department of Human Genetics	Samoan	Ryan L Minster	Pittsburgh	Pennsylvania	15260	United States of America
Weinstock, Joshua	University of Michigan	Biostatistics	IRC	Gonçalo Abecasis	Ann Arbor	Michigan	48109	United States of America
Weng, Lu-Chen	Massachusetts General Hospital		AFGen	Patrick Ellinor	Boston	Massachusetts	2114	United States of America

Wessel, Jennifer	Indiana University	Epidemiology	FHS, MESA	Jerome Rotter	Indianapolis	Indiana	46202	United States of America
Wessel, Jennifer	Indiana University	Epidemiology	FHS, MESA	Joanne Murabito	Indianapolis	Indiana	46202	United States of America
Willer, Cristen	University of Michigan	Internal Medicine	Analysis Program, IRC	Gonçalo Abecasis	Ann Arbor	Michigan	48109	United States of America
Williams, Kayleen	University of Washington	Biostatistics	MESA	Jerome Rotter	Seattle	Washington	98195	United States of America
Williams, L. Keoki	Henry Ford Health System		ATGC	Esteban Burchard	Detroit	Michigan	48202	United States of America
Williams, Scott	Case Western Reserve University		Africa6K	Sarah Tishkoff				
Wilson, Carla	Brigham & Women's Hospital		COPD	Ed Silverman	Boston	Massachusetts	2115	United States of America
Wilson, James	Beth Israel Deaconess Medical Center	Cardiology	Broad Institute and Beth Israel Proteomics Platform, JHS	Laura Raffield	Cambridge	Massachusetts	2139	United States of America
Wilson, James	Beth Israel Deaconess Medical Center	Cardiology	Broad Institute and Beth Israel Proteomics Platform, JHS	Robert Gerszten	Cambridge	Massachusetts	2139	United States of America
Winterkorn, Lara	New York Genome Center		New York Genome Center Genomics	Soren Germer	New York City	New York	10013	United States of America
Wong, Quenna	University of Washington				Seattle	Washington	98195	United States of America
Wu, Baojun	Henry Ford Health System	Department of Medicine	ATGC	Esteban Burchard	Detroit	Michigan	48202	United States of America
Wu, Joseph	Stanford University	Stanford Cardiovascular Institute	SCVI	Joseph Wu	Stanford	California	94305	United States of America
Xu, Huichun	University of Maryland		Amish	Braxton D. Mitchell	Baltimore	Maryland	21201	United States of America
Yanek, Lisa	Johns Hopkins University		GeneSTAR	Lisa Yanek	Baltimore	Maryland	21218	United States of America
Yang, Ivana	University of Colorado at Denver		IPF	David Schwartz	Denver	Colorado	80204	United States of America
Yu, Ketian	University of Michigan		IRC	Gonçalo Abecasis	Ann Arbor	Michigan	48109	United States of America
Zekavat, Seyedeh Maryam	Broad Institute		Broad Institute Genomics Platform	Stacey Gabriel	Cambridge	Massachusetts	2142	United States of America
Zhang, Yingze	University of Pittsburgh	Medicine	walk_PHaSST	Victor Gordeuk	Pittsburgh	Pennsylvania	15260	United States of America
Zhao, Snow Xueyan	National Jewish Health		IPF	David Schwartz	Denver	Colorado	80206	United States of America
Zhao, Wei	University of Michigan	Department of Epidemiology	HyperGEN_GENOA	Donna K Arnett	Ann Arbor	Michigan	48109	United States of America
Zhu, Xiaofeng	Case Western Reserve University	Department of Population and Quantitative Health Sciences	CFS	Susan Redline	Cleveland	Ohio	44106	United States of America
Ziv, Elad	University of California, San Francisco	Medicine	ATGC	Esteban Burchard	San Francisco	California	94143	United States of America
Zody, Michael	New York Genome Center		New York Genome Center Genomics	Soren Germer	New York	New York	10013	United States of America
